# MLKL trafficking and accumulation at the plasma membrane control the kinetics and threshold for necroptosis

**DOI:** 10.1038/s41467-020-16887-1

**Published:** 2020-06-19

**Authors:** Andre L. Samson, Ying Zhang, Niall D. Geoghegan, Xavier J. Gavin, Katherine A. Davies, Michael J. Mlodzianoski, Lachlan W. Whitehead, Daniel Frank, Sarah E. Garnish, Cheree Fitzgibbon, Anne Hempel, Samuel N. Young, Annette V. Jacobsen, Wayne Cawthorne, Emma J. Petrie, Maree C. Faux, Kristy Shield-Artin, Najoua Lalaoui, Joanne M. Hildebrand, John Silke, Kelly L. Rogers, Guillaume Lessene, Edwin D. Hawkins, James M. Murphy

**Affiliations:** 1 The Walter and Eliza Hall Institute of Medical Research, Parkville, VIC Australia; 2 Department of Medical Biology, The University of Melbourne, Parkville, VIC Australia; 3 Department of Pharmacology and Therapeutics, The University of Melbourne, Parkville, VIC Australia

**Keywords:** Biochemistry, Necroptosis

## Abstract

Mixed lineage kinase domain-like (MLKL) is the terminal protein in the pro-inflammatory necroptotic cell death program. RIPK3-mediated phosphorylation is thought to initiate MLKL oligomerization, membrane translocation and membrane disruption, although the precise choreography of events is incompletely understood. Here, we use single-cell imaging approaches to map the chronology of endogenous human MLKL activation during necroptosis. During the effector phase of necroptosis, we observe that phosphorylated MLKL assembles into higher order species on presumed cytoplasmic necrosomes. Subsequently, MLKL co-traffics with tight junction proteins to the cell periphery via Golgi-microtubule-actin-dependent mechanisms. MLKL and tight junction proteins then steadily co-accumulate at the plasma membrane as heterogeneous micron-sized hotspots. Our studies identify MLKL trafficking and plasma membrane accumulation as crucial necroptosis checkpoints. Furthermore, the accumulation of phosphorylated MLKL at intercellular junctions accelerates necroptosis between neighbouring cells, which may be relevant to inflammatory bowel disease and other necroptosis-mediated enteropathies.

## Abstract (web summary)

Mixed lineage kinase domain-like (MLKL) is the terminal protein in the pro-inflammatory necroptotic cell death program. Here the authors show that MLKL trafficking and plasma membrane accumulation are crucial necroptosis checkpoints, and that accumulation of phosphorylated MLKL at intercellular junctions promotes necroptosis.

## Introduction

Necroptosis is a caspase-independent, lytic form of programmed cell death^1–7^, which ancestrally has been implicated in host defense^3,8–11^. Necroptosis is also thought to participate in multiple human pathologies including ischemia-reperfusion injuries^12,13^, degenerative diseases^14^ and inflammatory diseases^12,15–20^. Necroptosis is known to be induced downstream of death, Toll-like and interferon receptor activation by their cognate ligands. Among these, TNF activation of TNF receptor 1 is the best-understood initiator of necroptotic signaling, and is a widely-used laboratory stimulus. Cells are predisposed to undergo death signaling when Receptor-interacting serine/threonine-protein kinase (RIPK)-1 is unable to be ubiquitylated by E3 ligases, such as the cellular inhibitor of apoptosis proteins (cIAPs). In scenarios where the apoptotic effector protease, Caspase-8, is inhibited chemically^1^ or by proteins, such as c-FLIP_s_^21^, RIPK1 can assemble with RIPK3 into a high molecular weight oligomeric complex known as the necrosome. Within the necrosome, RIPK3 in turn is activated by autophosphorylation, and subsequently phosphorylates the cytosolic pseudokinase, mixed lineage kinase domain-like (MLKL)^3–6,22,23^. While the precise mechanism of MLKL activation likely differs between species^24–26^, RIPK3-mediated phosphorylation of MLKL at the necrosome precedes the translocation of MLKL to the plasma membrane, where it causes cell death by compromising membrane integrity^23,27,28^.

Although our understanding of the necroptotic pathway is still in its infancy, much attention has been paid to the upstream events that govern MLKL activation and the terminal event whereby MLKL disrupts membrane integrity. By comparison, the molecular mechanisms of intervening steps, such as MLKL’s translocation to the plasma membrane to trigger necroptosis, are poorly understood. To date, proteins of the ESCRT-III complex, Flotillins and Alix:Syntenin-1 have been reported to regulate MLKL localization^29–31^, mostly in the context of endosomal compartmentalization or exosome shedding of activated MLKL to negate cell death. Which, if any, of these pathways regulate MLKL trafficking to the plasma membrane, where membrane integrity is disrupted to induce cell death, remains unclear. To address this gap in knowledge, we devised an immunofluorescence-based technique to temporally track and quantify the activation and translocation of endogenous human MLKL during necroptosis at the single-cell level. Using this approach, we have defined the rate-limiting mechanisms that control the trafficking of MLKL from the necrosome to the plasma membrane. We identified a Golgi-microtubule-actin-dependent pathway as a crucial regulatory step during the effector phase of necroptotic signaling, which controls MLKL translocation to the plasma membrane in epithelial and hematopoietic cell lines. Interestingly, MLKL shares the same trafficking pathway as the tight junction-associated protein, Zonula Occludens-1 (ZO-1), and co-accumulates with tight junction proteins as large hotspots at the plasma membrane during epithelial cell necroptosis. Furthermore, once at the plasma membrane, we find that stabilizing the peri-junctional organization of ZO-1 potently counteracts the ability of MLKL to cause epithelial cell necroptosis. Conversely, destabilizing the peri-junctional organization of ZO-1 sensitizes epithelial cells to necroptosis. Hence, the trafficking and membranolytic threshold for MLKL is mechanistically linked to tight junction proteins during epithelial cell necroptosis. Prompted by this interplay, we show that peri-junctional damage to the plasma membrane occurs during epithelial cell necroptosis, which in turn propagates membrane damage and accelerates the death of neighboring cells.

Our findings uncover two new checkpoints in necroptosis: one is a trafficking mechanism that regulates the kinetics of necroptosis; and the other controls the amount of MLKL needed to disrupt plasma membranes, which in turn facilitates neighboring cell death in a cell-extrinsic manner. These post-necrosomal checkpoints represent additional targets for therapeutic intervention in the necroptosis pathway.

## Results

### Single-cell methodology to study MLKL activation

**Fig. 1:**
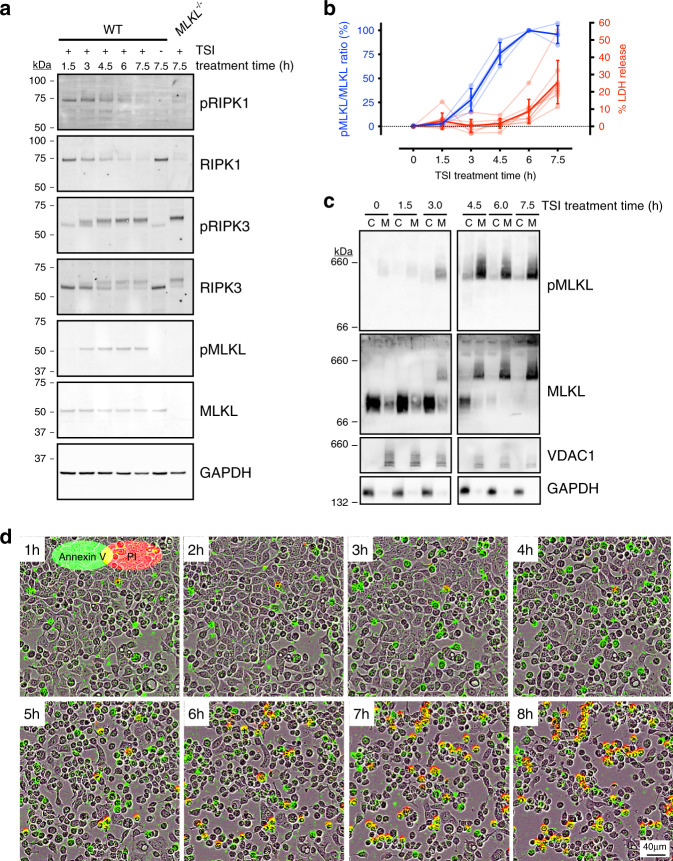
A temporal gap occurs between MLKL activation and necroptosis. Wild-type (WT) and *MLKL*^*−/−*^ HT29 cells were treated with media alone or the necroptotic stimuli, TNF and Smac-mimetic and pan-caspase inhibitor, IDN-6556, (TSI) for the indicated time period and chronological RIPK1, RIPK3, and MLKL phosphorylation (**a**), plasma membrane lysis (**b**), MLKL oligomerization and membrane-association (**c**), and plasma membrane damage with cell death (**d**) were measured. **a** Whole-cell lysates were fractioned by SDS-PAGE and immunoblotted to determine the extent of RIPK1, RIPK3 and MLKL phosphorylation over time. The displayed immunoblots are representative of *n* = 2 independent experiments for RIPK1/3 and *n* = 4 independent experiments for MLKL. **b** Left-hand y-axis plots the ratio of the pMLKL and MLKL immunoblot signals (relative to the *t* = 6 h time point value) across *n* = 4 independent experiments. Mean ± SD. Right-hand *y*-axis plots the extracellular release of lactate dehydrogenase (LDH) as an index of plasma membrane lysis (relative to detergent-treated cells) across *n* = 9 independent experiments. Mean ± SEM. All data are included in the Source Data file. **c** Subcellular fractions of the cytosol (C) and membrane (M) were subjected to Blue Native-PAGE and immunoblotted to assess MLKL phosphorylation, oligomerization and membrane-association. Fractionation was verified by probing for the membrane protein, voltage-dependent anion channel 1 (VDAC1), and the cytosolic protein, glyceraldehyde-3-phosphosphate dehydrogenase (GAPDH). Data are representative of *n* = 2 independent experiments. **d** Sequential time-lapse micrographs of Annexin V binding (Annexin V; green) and propidium iodide-uptake (PI; red) as indices of plasma membrane damage and cell death, respectively. Micrographs are representative of *n* = 4 independent experiments.

To gauge the contribution of regulatory mechanisms downstream of necrosome assembly and RIPK3 activation, we compared the kinetics of RIPK1, RIPK3 and MLKL phosphorylation and plasma membrane lysis in multiple human epithelial cell lines treated with the necroptosis-inducing stimulus TNF, Smac-mimetic and IDN-6556 (hereafter referred to as TSI; Fig. 1a, b and Supplementary Fig. 1). Immunoblot analyses showed that RIPK1 was rapidly phosphorylated at S166 (pRIPK1) within 1.5 h of TSI-treatment (Fig. 1a). Subsequently, RIPK3 phosphorylation at S227 (pRIPK3) and MLKL phosphorylation at S358 (pMLKL) was evident after 3 h of TSI-treatment and increased until 6 h post-TSI treatment (Fig. 1a, b). Notably, all cell lines exhibited a temporal gap between the emergence of pMLKL and the onset of plasma membrane lysis (as measured by LDH release), which was most pronounced in the HT29 colorectal cancer cell line (Fig. 1a, b and Supplementary Fig. 1). Emergence of pMLKL was mirrored by the onset of pMLKL oligomerization and membrane association (Fig. 1c), which all preceded cell death by more than 3 h (Fig. 1d) in HT29 cells following TSI-treatment. This temporal gap between the hallmarks of MLKL activation (namely MLKL phosphorylation, oligomerization and membrane association) and subsequent cell death has been noted before^32^; suggesting that rate-limiting mechanisms exist for necroptosis downstream of MLKL recruitment to, and activation within, the necrosome.

**Fig. 2:**
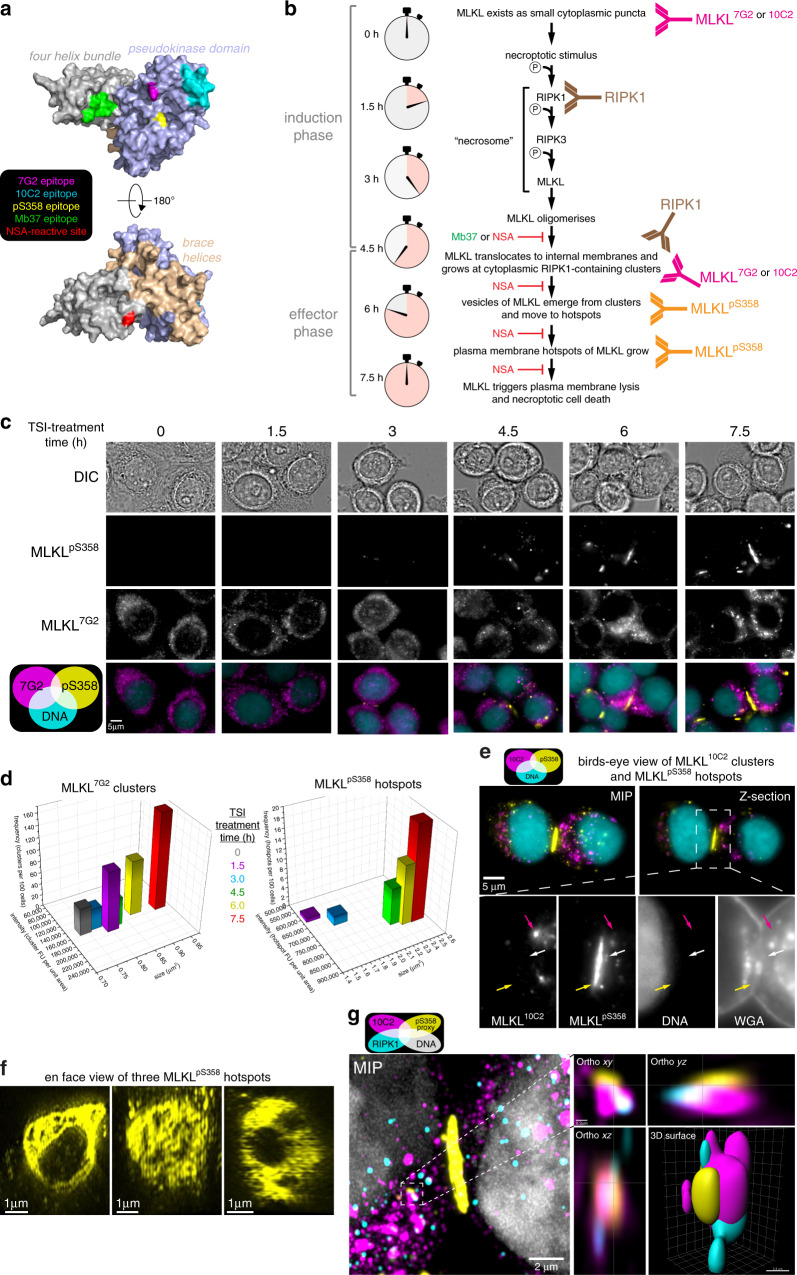
Death coincides with necrosomal and plasma membrane hotspot MLKL accumulation. **a** Homology model of full-length human MLKL^66^ highlighting the 7G2, 10C2 and pS358 antibody and Monobody 37 (Mb37)^37^ epitopes, and the target of necrosulfonamide (NSA), C86^6^. **b** Model for immunofluorescent detection of the stages of RIPK1 and MLKL activation. **c–d** TSI-treated HT29 cells stained for MLKL^7G2^, MLKL^pS358^ and DNA (Hoechst). Imaged by epifluorescence and differential interference contrast (DIC) microscopy. Representative of *n* = 4 independent experiments. **c** MLKL^7G2^ clusters, MLKL^pS358^ hotspots and cell lysis emerge >4.5 h post-TSI treatment. **d** Mean frequency, intensity and size of clusters and hotspots during TSI-induced necroptosis from one experiment (*N* = 1258-1973 cells per timepoint). **e** 3D-SIM maximum intensity projection (MIP), Z-section and zoomed micrographs of TSI-treated HT29 cells revealed MLKL^pS358^ puncta (white arrow) located basally to a MLKL^pS358^ hotspot. The hotspot colocalizes with the Wheat germ agglutinin (WGA)-stained plasma membrane of an intercellular junction (yellow arrow) apical to MLKL^10C2^ clusters (magenta arrow). **f** En face view of three different MLKL^pS358^ hotspots of heterogeneous substructure. **g** TSI-treated HT29 cells were stained for MLKL^10C2^, RIPK1, ZO-1 (a proxy for the MLKL^pS358^ immunosignal; refer to Fig. 8a), and DNA (Hoechst), and imaged by 3D Airyscan microscopy (representative of *n* = 3 independent experiments).

We chose the HT29 line, in the first instance, to investigate the post-necrosomal regulation of MLKL. Current methods for assessing cellular MLKL activation provide critical insight into the induction phase of necroptosis (i.e., 1.5–4.5 h TSI-treatment), but are less informative during the effector phase of necroptosis (e.g., ≥4.5 h TSI-treatment; Fig. 1). Thus, we developed a quantitative single-cell approach to measure the activation and translocation of endogenous human MLKL. To this end, we generated two monoclonal antibodies – the 7G2 and 10C2 clones – that recognize distinct epitopes within human MLKL (Fig. 2a and Supplementary Fig. 2a, b). We used these antibodies, together with an established anti-MLKL^pS358^ antibody^23^, to optimize the immunofluorescent detection of MLKL. Immunofluorescence using either the 7G2 or 10C2 antibodies showed that MLKL resides in small cytoplasmic puncta in the basal state, which then coalesce into larger cytoplasmic clusters during the effector phase of necroptosis (Fig. 2b–f). These clusters of MLKL grow around congregations of RIPK1 (Fig. 2g and Supplementary Movie 1), indicating that they are necrosome-related. Consistent with this finding, small puncta of MLKL^pS358^ originated from these cytoplasmic necrosomal clusters (Fig. 2e–g) and progressively accumulated as hotspots of MLKL^pS358^ at the plasma membrane (Fig. 2b–g). The formation of MLKL clusters and hotspots appears to be a hallmark of necroptosis as these structures were observed in two epithelial cell lines treated with different necroptotic stimuli, but not during extrinsic or intrinsic apoptosis (Supplementary Fig. 2c–f). As expected, loss of RIPK1, RIPK3 or MLKL completely abrogated formation of hotspots and clusters (Supplementary Fig. 2d).

**Fig. 3:**
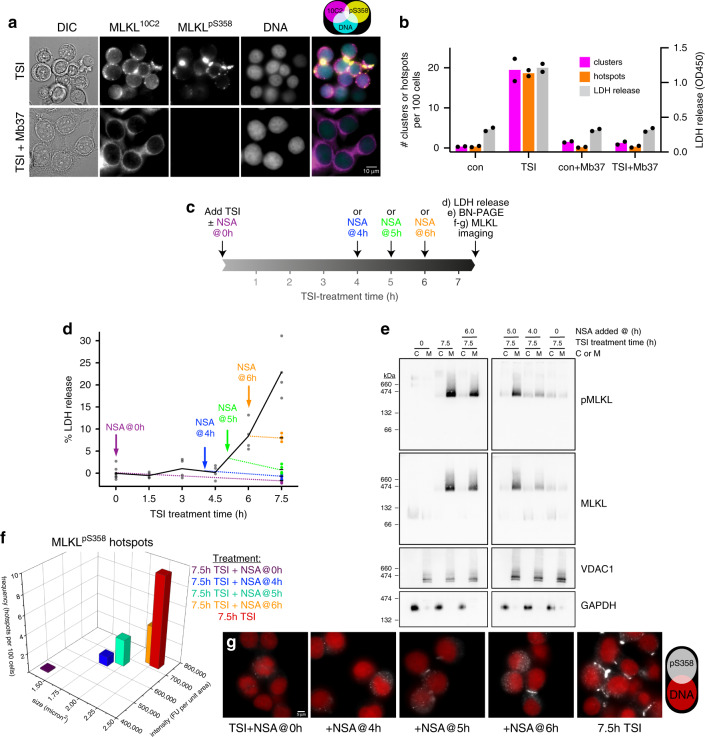
Necroptosis relies on exceeding a threshold level of plasma membrane pMLKL. **a** Images of TSI-treated HT29 cells expressing Mb37 stained for MLKL^10C2^, MLKL^pS358^ and DNA (Hoechst). **b** Mean frequency of clusters and hotspots from *n* = 2 independent experiments (*N* > 1500 cells per group) with raw lactate dehydrogenase (LDH) release values taken from the same samples. **c**–**g** Necrosulfonamide (NSA) was added to HT29 cells together with TNF, Smac mimetic and IDN-6556 (TSI) or 4–6 h after TSI addition. Plasma membrane lysis (LDH release; line shows mean of 4 biological replicates indicated as dots; data representative of *n* = 3 independent experiments) (**d**), MLKL activation (**e**) and hotspots (**f, g**) were measured after 7.5 h of TSI-treatment. **e** Cytosol (C) and membrane (M) fractions resolved by Blue Native-PAGE and immunoblotted for MLKL and MLKL^pS358^. Uncropped blots are included as Source Data. Fractionation was verified by probing for voltage-dependent anion channel 1 (VDAC1; membrane), glyceraldehyde-3-phosphosphate dehydrogenase GAPDH (cytosol); representative of *n* = 2 independent experiments. **f** Mean frequency, intensity and size of hotspots from one experiment (*N* = 1309-1740 cells per group); representative of *n* = 3 independent experiments.

Some unexpected features of the clusters and hotspots were noted. Firstly, rather than associating uniformly with the plasma membrane, MLKL^pS358^ typically accumulates as 1–2 hotspots per cell in HT29 and HCC2998 cultures undergoing necroptosis (Fig. 2 and Supplementary Fig. 2c–f). These hotspots were ~0.5–3 μm in diameter, preferentially located to intercellular junctions, and had an irregular ring-like architecture containing heterogeneous substructure (Fig. 2e, f and Supplementary Movie 2). This architecture is distinct from other membrane-associated executioner complexes, such as apoptotic pore complexes, the Gasdermin family and membrane attack complex/perforin family^33–36^, and does not support the idea that MLKL forms regularly-structured membrane pores. Secondly, only a modest degree of overlap was observed between the MLKL^10C2^ and MLKL^pS358^ immunosignals (Supplementary Fig. 2g), which suggests that these immunosignals reflect different chronological states of MLKL activation (Fig. 2b). To investigate this possibility, we employed two inhibitors that act downstream of MLKL phosphorylation: the synthetic protein ligand, Monobody 37 (Mb37)^37^, and the compound, necrosulfonamide (NSA)^6^. Mb37 binds the killer four-helix bundle domain (Fig. 2a) of human MLKL to block membrane translocation and cell death, but does not inhibit MLKL phosphorylation or oligomerization^37^. Like Mb37, NSA does not block MLKL phosphorylation, but is instead thought to prevent MLKL from associating with membranes^23,38^. Indeed, phosphorylated MLKL could be readily detected by immunoblot in HT29 cells treated with necroptotic stimuli in the presence of Mb37 (as shown in ref. ^37^) or NSA (Supplementary Fig. 1). Despite this, neither clusters nor hotspots of MLKL were observed in the presence of Mb37 (Fig. 3a, b) or NSA (Fig. 3c–g) confirming that these MLKL-containing structures are formed downstream of MLKL phosphorylation, oligomerisation and membrane-association (Fig. 2b).

We propose that membrane-associated MLKL^10C2^ clusters accumulate around RIPK1-containing necrosomes in the cytosol during the effector phase of necroptosis (Fig. 2b, g). These clusters resemble the previously described RIPK3-containing puncta that form during necroptosis^6^. We further propose that vesicle-associated MLKL^pS358^ then emerges from these clusters and traffics to the cell periphery where it accumulates as hotspots at the plasma membrane (Fig. 2b). The increases in the occurrence/size of the MLKL^10C2^ clusters and the occurrence/size/intensity of the MLKL^pS358^ hotspots mirrors plasma membrane lysis (compare Fig. 2d and Supplementary Fig. 2h with Fig. 1b). In line with this conclusion, adding NSA during the effector phase of necroptosis (i.e., ≥5 h of TSI-treatment in HT29 cells when oligomeric pMLKL is already membrane-associated on vesicles) not only profoundly reduced cell death (Fig. 3d), but also markedly reduced the size, intensity and occurrence of MLKL^pS358^ hotspots on the plasma membrane (Fig. 3f, g). Hence, our immunofluorescence protocol provides quantitative insights into intracellular trafficking events that occur downstream of the phosphorylation, oligomerization and membrane-translocation of MLKL.

### MLKL hotspots accelerate necroptosis in neighboring cells

**Fig. 4:**
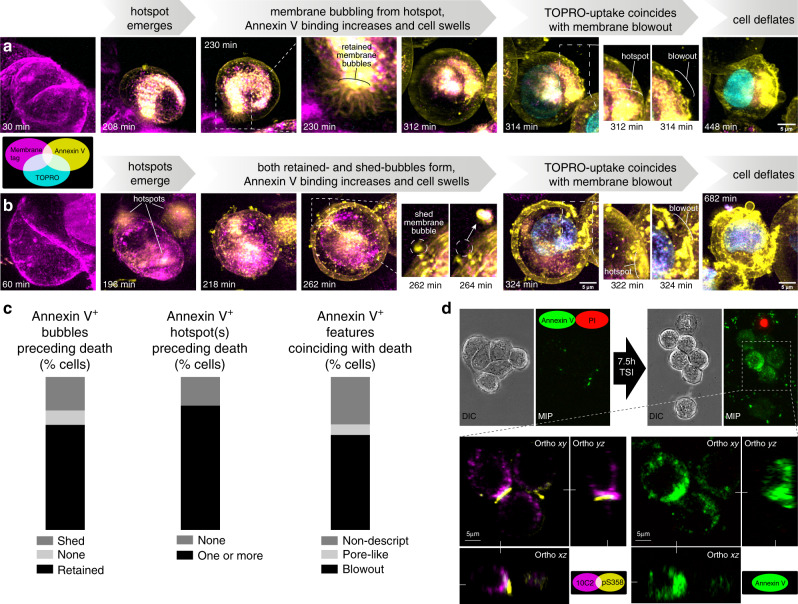
Membrane damage at MLKL hotspots promotes cell death. **a**–**c** Time-lapse lattice lightsheet microscopy of TSI-treated HT29 cells expressing mTagRFP-Membrane-1 fusion protein (magenta; plasma membrane) with Annexin V-binding (yellow; membrane damage) and TOPRO-uptake (cyan; cell death). Micrographs are maximum intensity projections (MIP). Plots in **c** show the percentage of cells that shed or retained bubbles or formed hotspots preceding death, or featured membrane blowout or pore-like structures contemporaneously with death. Data from *N* = 32 cells across *n* = 4 independent experiments for events preceding death and *N* = 29 across *n* = 3 independent experiments for events coinciding with death. **d** HT29 cells were TSI-treated and Annexin V-binding (Annexin V; green) and propidium iodide-uptake (PI; red) were measured via time-lapse DIC and 3D confocal microscopy. After 7.5 h of TSI-treatment, cells were immunostained for MLKL^10C2^ and MLKL^pS358^. Annexin V-binding during TSI-treatment in MIP; orthogonal (Ortho) projections show intense Annexin V-binding proximal to a MLKL^pS358^ hotspot (representative of *N* > 30 cells across 3 separate fields from one experiment).

To assess whether focal accumulation of MLKL^pS358^ at hotspots affects plasma membrane integrity, we treated HT29 cells with necroptotic stimuli and visualized the resulting plasma membrane damage reflected by Annexin V binding to externalized phosphatidylserine, and cell death via TOPRO3-uptake using lattice light sheet microscopy. Akin to the accumulation of MLKL^pS358^ at the plasma membrane (Fig. 2a–c), sites of Annexin V binding first appeared 3–4.5 h after the induction of necroptosis (Fig. 4a–c and Supplementary Movie 3). These sites of Annexin V binding increased in size over time, were often situated near ongoing/former points of cell-to-cell contact, and persisted after death. Notably, Annexin V^+^ sites appeared within the immediate vicinity of MLKL^pS358^ hotspots on the plasma membrane during necroptosis (Fig. 4d and Supplementary Movie 4), raising the prospect that MLKL^pS358^ hotspots correlate with sites of membrane damage during epithelial cell necroptosis. Additionally, Annexin V^+^ membrane protrusions (previously described as bubbles^29^), were observed 1.5–4.5 h after the induction of necroptosis (Fig. 4a, b). These protrusions decreased in number as cells swelled near to time-of-death; they were heterogenous in size, shape, Annexin V binding intensity, and whether they were shed or retained by the cell (Fig. 4a–c). In addition, the protrusions did not necessarily appear at the same location as the Annexin V^+^ hotspots (Fig. 4a–c). Interestingly, HT29 cells died 1–3 h after the appearance of Annexin V^+^ hotspots, with a cataclysmic plasma membrane disruption event at a single site, which we term “membrane blowout”, being the most common feature coinciding with death (Fig. 4a–c). Collectively, these observations are consistent with our hypothesis that pMLKL accumulates and damages the plasma membrane at hotspots in epithelial cells, which eventually triggers local membrane blowout and necroptotic death.

**Fig. 5:**
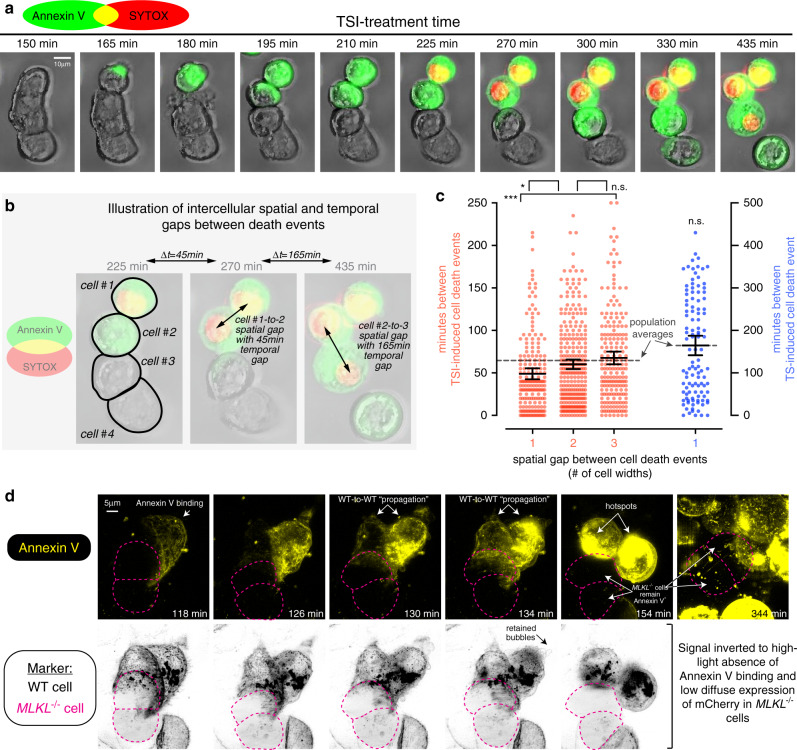
Necroptosis is accelerated between junctioned epithelial cells. **a** HT29 cells were TNF, Smac mimetic and IDN-6556 (TSI)-treated, then Annexin V-binding (green) and SYTOX Green-uptake (red) were imaged via time-lapse epifluorescence microscopy. **b, c** HT29 cells were treated with TSI (necroptotic stimulus) or TS (apoptotic stimulus), then propidium iodide-uptake (death) and subsequent movement of dead cells was tracked over time using the IncuCyte S3 System. **d** Each dot represents the spatiotemporal gap between two cell death events. Mean ± 95% confidence intervals; dashed line represents the average spatiotemporal gap between all cell death events across the population; ^**n.s**.^*p* = 0.2223, *p = 0.0422 and ***p = 0.0006 via one-way ANOVA with Tukey’s correction for multiple comparisons. For TSI-treated HT29 cells: *N* = 188, 289, 298 gaps for the respective 1, 2, 3-cell-width groups and *N* = 22676 gaps for the whole population. For TS-treated HT29 cells: *N* = 110 gaps for 1-cell-width group and *N* = 17838 gaps for the whole population. **e** Co-cultures of TSI-treated wild-type (expressing mTagRFP-Membrane-1 fusion protein) and *MLKL*^*−/−*^ (diffuse expression of mCherry) HT29 cells were imaged via time-lapse lattice light sheet microscopy (LLSM) with staining for Annexin V-binding (top row; yellow signal) and markers (bottom row; same as top row but with inverted signal). MIP micrographs are representative of *N* = 7 *MLKL*^*−/−*^ cells across *n* = 3 independent experiments. TSI-treatment times are shown; magenta lines demarcate *MLKL*^*−/−*^ cell boundaries.

Given that MLKL-mediated membrane damage preferentially occurs at intercellular junctions, we next considered whether necroptosis could be communicated to neighboring cells and influence their susceptibility to necroptotic death. To address this, we used widefield time-lapse imaging to track plasma membrane damage and cell death events across a large population of HT29 cells undergoing TNF-induced necroptosis. These experiments showed that membrane damage, as indicated by phosphatidylserine exposure, could propagate from one cell to its abutting neighbor, like a chain reaction (Fig. 5a). Despite the onset of death after propagation of membrane damage to a neighboring cell being highly variable, large-scale analysis showed that adjacent HT29 cells died via necroptosis ~25% faster than their non-adjacent counterparts (Fig. 5a–c). Importantly, no promotion of death occurred between neighboring HT29 cells undergoing TNF-induced apoptosis, which is an MLKL-independent form of cell death (Fig. 5b, c). To ascertain whether necroptosis of one cell was sufficient to damage neighboring cells, we treated co-cultures of wild-type and *MLKL*^*−/−*^ HT29 cells with necroptotic stimuli and used high spatio-temporal resolution lattice light sheet microscopy to visualize membrane damage over time. Unlike the propagation of membrane damage between neighboring wild-type HT29 cells, there was no evidence for the propagation of Annexin V binding from a wild-type necroptotic cell to a neighboring *MLKL*^*−/−*^ cell (Fig. 5d). Moreover, in these co-culture experiments, no *MLKL*^*−/−*^ HT29 cells (*N* = 7 cells from *n* = 3 independent experiments) died in response to necroptotic stimuli. Together, these data suggest that junctional accumulation of MLKL accelerates death of adjacent epithelial cells, but only when both neighboring cells are primed to undergo necroptosis. We propose that this is a junction-related ancillary mechanism for accelerating necroptosis between neighboring cells.

### MLKL is actively trafficked to the plasma membrane

**Fig. 6:**
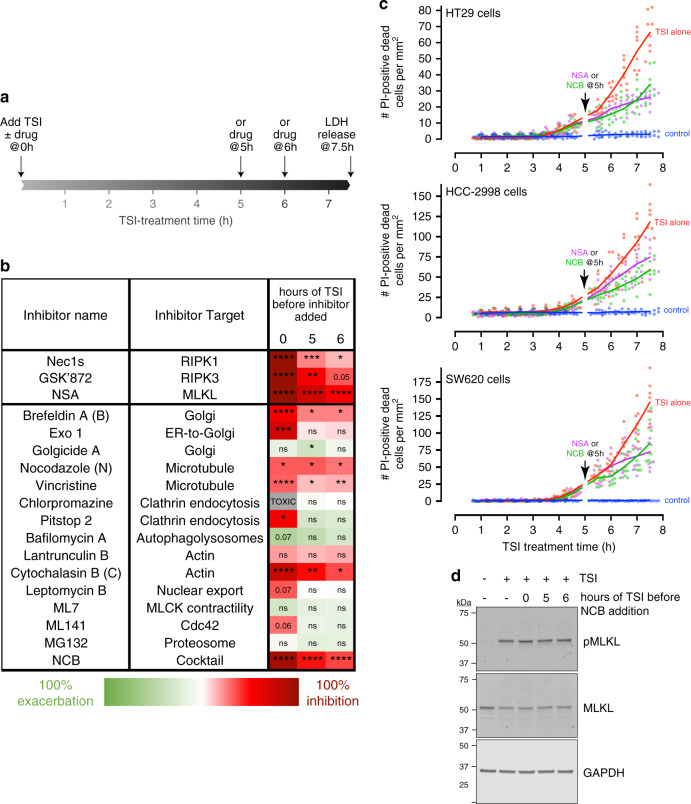
Intracellular trafficking mechanisms govern necroptotic death kinetics. **a, b, d** The indicated necroptosis-, trafficking- or organellar-inhibitors were added to HT29 cells with TNF, Smac mimetic and IDN-6556 (TSI) or 5–6 h after TSI-treatment. Plasma membrane lysis (**b**) and MLKL phosphorylation (**d**) were examined 7.5 h post-TSI treatment (as depicted schematically in **a**). **b** Chart summarizes the impact of inhibitors on lactate dehydrogenase (LDH) release (relative to TSI-treated cells). Color coding indicates mean values across *n* = 3–4 independent experiments; ‘0% inhibition’ indicates no LDH release above untreated cells and ‘100% exacerbation’ indicates a doubling of LDH release above TSI-treated cells. Chlorpromazine was toxic upon prolonged exposure (gray). *p < 0.05, **p < 0.01, ***p < 0.001 and ****p < 0.0001 and p-values between 0.05 and 0.1 are shown as determined by one-way ANOVA with Dunnett’s correction for multiple comparisons. Independent datapoints underlying (**b**) are shown in Supplementary Fig. 3a. **c** HT29, HCC2998 and SW620 cells were left untreated (control) or TSI-treated. Necrosulfonamide (NSA) or Nocodazole, Cytochalasin B, Brefeldin A (NCB) was added after 5 h of TSI-treatment. Propidium iodide (PI)^+^ (dead) cells per mm^2^ over time were measured using an IncuCyte S3 System (mean of *N* = 3 fields with >600 cells per group from one experiment with the underlying biological replicates shown as dots; data representative of *n* = 2 independent experiments). **d** Immunoblots of whole-cell lysates indicate that NCB does not affect TSI-induced MLKL phosphorylation (representative of *n* = 2 independent experiments).

Having ascertained that hotspots are a primary site of membrane damage during necroptosis, we postulated that the temporal gap between MLKL phosphorylation and cell death reflects the time needed for MLKL to traffic from the necrosome to these hotspots. To test this idea, we examined the role of various trafficking mechanisms across the induction and effector phases of necroptosis using a range of inhibitors (Fig. 6a). We were primarily interested in inhibitors that interfered with steps downstream of MLKL phosphorylation, and were thus capable of blocking cell death when added during either the induction or effector phases of necroptosis. Interestingly, we observed that inhibitors that destabilize trafficking along the Golgi, microtubule, and actin networks attenuated plasma membrane lysis when added during either the induction or effector phases of necroptosis (Fig. 6b and Supplementary Fig. 3a). Inhibiting these trafficking pathways with a cocktail of Nocodazole, Cytochalasin B and Brefeldin A (NCB) was a particularly potent approach to suppress plasma membrane lysis at any timepoint during necroptosis (Fig. 6a, b and Supplementary Fig. 3a). Importantly, addition of either NSA or NCB five hours after TSI-treatment, when oligomeric MLKL^pS358^ had already begun to accumulate at the plasma membrane, inhibited subsequent necroptotic death of three human epithelial cell lines: HT29, HCC2998 and SW620 (Fig. 6c). NCB also attenuated TNF-induced necroptosis in the mouse YAMC epithelial cell line (Supplementary Fig. 3b).

It is salient that addition of NCB during the effector phase of necroptosis immediately stalled subsequent cell death (Fig. 6c). This suggests that NCB may interfere with pMLKL trafficking and thereby prevent the threshold for plasma membrane lysis from being reached. By comparison, NCB did not reduce the onset of plasma membrane damage or cell death during the MLKL-independent processes of extrinsic- or intrinsic- apoptosis (Supplementary Fig. 3c). Moreover, NCB did not alter MLKL phosphorylation (as measured by immunoblot analyses; Fig. 6d) or prevent MLKL oligomerization and membrane translocation (as assessed by Blue-Native PAGE; Supplementary Fig. 3d) during necroptosis in HT29 cells. Collectively, these observations are consistent with the notion that NCB interrupts the trafficking of MLKL from the necrosome to the plasma membrane, rather than influencing MLKL activation at the necrosome or by affecting mechanisms that generically influence plasma membrane integrity and cell death.

**Fig. 7:**
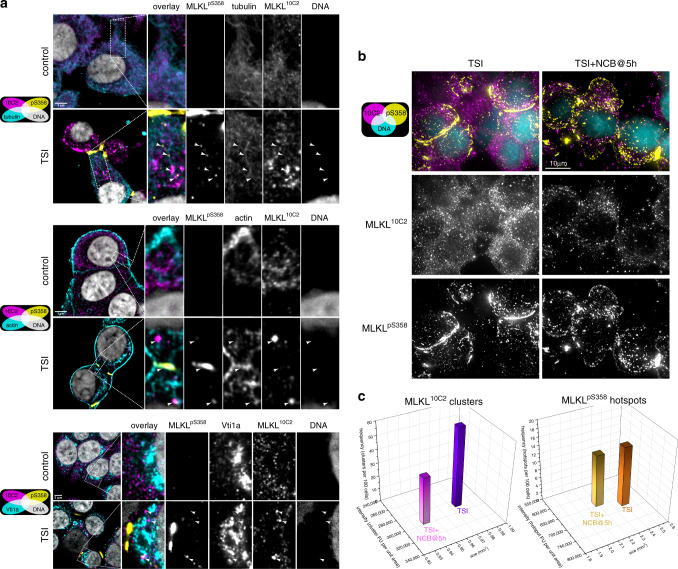
Microtubule-actin-Golgi machinery traffics MLKL to the plasma membrane. **a** HT29 cells were left untreated (control) or TNF, Smac mimetic and IDN-6556 (TSI)-treated for 7.5 h, then immunostained as noted and imaged by 3D Airyscan microscopy. Micrographs are single Z-sections. Arrowheads indicate MLKL^10C2^ clusters or MLKL^pS358^ vesicles or MLKL^pS358^ hotspots that colocalize with actin or tubulin filaments, and show negligible colocalization between MLKL^10C2/pS358^ and the Golgi marker, Vti1a, was observed. Micrographs are representative of *n* = 2 independent experiments. **b–c** Nocodazole, Cytochalasin B, Brefeldin A (NCB) was added to HT29 cells after 5 h of TSI-treatment. After 7.5 h of TSI-treatment, cells were stained for MLKL^10C2^, MLKL^pS358^ and DNA (Hoechst) and imaged via deconvolved 3D widefield (**b**) or epifluorescence (**c**) microscopy (representative of *n* = 3 independent experiments). **c** Mean frequency, intensity and size of clusters and hotspots across *n* = 3 independent experiments (*N* = 2830 and 2181 cells for the TSI and TSI + NCB@5 h groups, respectively). Independent datapoints in **c** are shown in Supplementary Fig. 3f.

We next examined the physical proximity of MLKL to components of NCB-sensitive trafficking pathways in HT29 cells undergoing necroptosis. We observed no substantive overlap between MLKL and the Golgi-marker Vti1a (Fig. 7a). In contrast, a substantial proportion of MLKL^10C2^ clusters and MLKL^pS358^ vesicles were positioned along tubulin and actin filaments running from the nucleus to MLKL^p358^ hotspots at the plasma membrane (Fig. 7a). Moreover, these MLKL^p358^ hotspots co-stained for the presence of cortical actin (Fig. 7a). These data are consistent with MLKL translocating in a polarized manner from cytoplasmic necrosomes along actin and tubulin filaments to the plasma membrane.

Notably, the described trafficking pathway for MLKL is not epithelial cell-specific, as NCB also slowed the rate of TNF-induced necroptosis in the Molt-4 (human acute T-lymphoblastic leukemia) and U937 (human monocytic lymphoma) cell lines (Supplementary Fig. 3e). NCB, however, did not markedly alter necroptotic cell death in the Colo-205 (human colorectal adenocarcinoma) or RPMI-8226 (human multiple myeloma line) cell lines (Supplementary Fig. 3e). Thus, a NCB-sensitive trafficking mechanism for MLKL is employed by many, but not all, cell types during necroptosis.

Unlike NSA, NCB does not prevent MLKL from associating with membranes, as MLKL clusters and hotspots were still readily detectable by immunofluorescence in HT29 cells that had been co-treated with TSI and NCB (Fig. 7b). Quantitation showed that the addition of NCB during necroptosis decreased the size of MLKL clusters and hotspots (Fig. 7c and Supplementary Fig. 3f). As the formation of clusters and hotspots occurs downstream of MLKL phosphorylation, these findings suggest that NCB slows both the emergence of membrane-associated MLKL^10C2^ and MLKL^pS358^ from the necrosome and its subsequent movement towards plasma membrane hotspots. NCB also depolarized the trafficking of MLKL and randomized the distribution of MLKL^pS358^ vesicles within the cell and at the plasma membrane (Fig. 7b). Together, our data illustrate that MLKL traffics from the necrosome to plasma membrane hotspots via Golgi-, actin- and microtubule-dependent mechanisms. This post-necrosomal trafficking event is a formerly-unrecognized rate-limiting checkpoint in necroptosis.

### Tight junction proteins and MLKL are co-trafficked

**Fig. 8:**
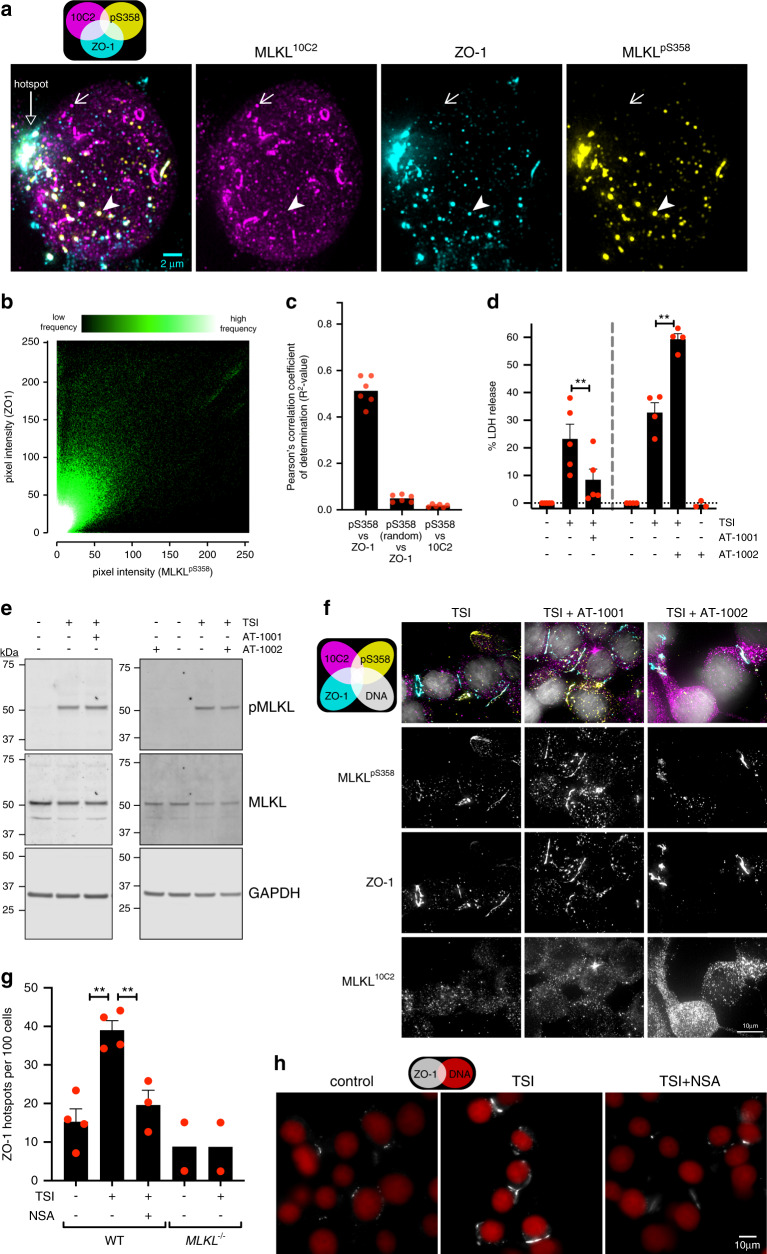
MLKL and ZO-1 trafficking crosstalk influences necroptotic lysis. **a, b** HT29 cells were TNF, Smac mimetic and IDN-6556 (TSI)-treated for 7.5 h, immunostained for MLKL^10C2^, ZO-1 and MLKL^pS358^ then imaged via deconvolved 3D widefield (**a**) or epifluorescence (**b**, **c**) microscopy. **a** Micrograph is a maximum intensity projection and representative of *n* = 5 independent experiments. Diagonal closed arrowhead indicates an intracellular MLKL^pS358^ vesicle that co-stains for ZO-1. Diagonal arrow indicates a MLKL^10C2^ cluster that does not co-stain for ZO-1 or MLKL^pS358^. **b** 2D histogram depicts the frequency of MLKL^pS358^ and ZO-1 immunosignal pixel co-intensities across *N* = 514 cells from *n* = 2 independent experiments. **c** Pearson’s correlation of MLKL^pS358^ and ZO-1 immunosignals. Two negative controls are shown (MLKL^pS358^ versus MLKL^10C2^ or randomly redistributed MLKL^pS358^ immunosignals). Graph shows the mean (with underlying datapoints as dots) from *n* = 6 independent fields and *N* = 514 cells. **d**–**f** HT29 cells were left untreated or treated with TSI in the presence of AT-1001 or AT-1002. Lactate dehydrogenase (LDH) release (**d**), MLKL phosphorylation (**e**) or MLKL^pS358^ hotspots (**f**) were assessed after 7.5 h of treatment. **d** Mean +SEM of LDH release from *n* = 3–5 independent experiments (dots indicate mean of *N* = 4 biological replicates from each underlying experiment). **p = 0.0030 via paired two-tailed t-test for the anti-necroptotic effect of AT-1001 and **p = 0.0046 via one-way ANOVA with Tukey’s post-hoc correction for the pro-necroptotic effect of AT-1002. **e** Whole-cell lysates were fractioned by SDS-PAGE and immunoblotted to determine if AT-1001 or AT-1002 alter the extent of TSI-induced MLKL phosphorylation. Data are representative of *n* = 2 independent experiments. **f** Cells were immunostained for MLKL^10C2^, ZO-1 and MLKL^pS358^ then imaged via deconvolved 3D-widefield. Micrographs are maximum intensity projections and representative of *n* = 2 experiments. **g, h** Wild-type and *MLKL*^*−/−*^ HT29 cells were treated with for 7.5 h with TSI and/or necrosulfonamide (NSA), then immunostained for ZO-1 and imaged via epifluorescence microscopy. Graph shows the number of ZO-1 hotspots across *n* = 3–4 independent experiments for wild-type (WT) and *n* = 2 for *MLKL*^*−/−*^ HT29 cells (*N* = 2126–4162 cells per group; mean + SEM; dots indicate mean of each experiment). **p = 0.0017 or **p = 0.0072 via paired one-way ANOVA with Tukey’s post-hoc correction. Micrographs are representative of *n* = 3–4 independent experiments.

To deduce the nature of the membrane-associated MLKL^pS358^ puncta and hotspots that form during necroptosis, we co-stained HT29 cells undergoing necroptosis for MLKL^pS358^ and various junction, organelle or microdomain markers. Negligible colocalization was observed between MLKL^pS358^ and markers of desmosomes (Desmoglein-2), adherens junctions (E-cadherin), endosomes and caveolae (Flotillin-1), mitochondria (TOMM20), lysosomes (LAMP2), proteasome (20S) or the chaperone HSP90^39^ (Supplementary Fig. 4a). However, colocalization was evident between MLKL^pS358^ and three tight junction markers: JAM-A, occludin and ZO-1 (Fig. 8a and Supplementary Fig. 4b). Colocalization between MLKL^pS358^ and tight junction markers was also observed in HCC2998 cells undergoing necroptosis (Supplementary Fig. 4c). Strikingly, ZO-1 colocalized with MLKL^pS358^ both at hotspots and intracellular puncta, suggesting that MLKL is co-trafficked with tight junction proteins to the plasma membrane during necroptosis in HT29 cells (Fig. 8a). Furthermore, this colocalization was quantitative in nature, with pixel-level proportionality observed between the MLKL^pS358^ and ZO-1 immunosignals (Fig. 8b, c). These data suggest that tight junction proteins and MLKL rely on the same trafficking pathways for membrane translocation during epithelial cell necroptosis.

Based on the compelling colocalization of ZO-1 and MLKL^pS358^ in epithelial cells, we further explored whether regulatory crosstalk might exist between tight junction proteins and necroptotic signaling. Tight junctions are megadalton complexes that physically link the plasma membranes of neighboring cells, limit paracellular transport and thus are critical for epithelial barrier function^40^. AT-1001 and AT-1002 are cell-impermeable peptides that indirectly modulate tight junctions. AT-1001 preserves the peri-junctional organization of ZO-1 and protects barrier integrity during epithelial challenge^41^. Conversely, AT-1002 is thought to disrupt the peri-junctional organization of ZO-1 and induce epithelial barrier dysfunction^41^. To address whether modulating tight junctions influences necroptotic signaling, we induced necroptosis in HT29 cells in the presence of AT-1001 or AT-1002. AT-1001 attenuated plasma membrane lysis by ~75%, presumably owing to membrane rigidification at hotspots thereby raising the threshold of pMLKL required for membrane perturbation. Conversely, AT-1002 increased plasma membrane lysis by ~90% in HT29 cells treated with a necroptotic stimulus (Fig. 8d), presumably due to AT-1002 compromising membrane stability at hotspots and thereby lowering the threshold for pMLKL-mediated disruption. AT-1001 also reduced the extent of necroptotic death in three other epithelial cell lines: human HCC2998 cells, human Colo-205 cells and mouse YAMC cells (Supplementary Fig. 4d–f). Importantly, immunoblot analyses show that AT-1001 and AT-1002 did not alter TSI-induced MLKL phosphorylation, suggesting that these peptides act upon a post-necrosomal checkpoint (Fig. 8e). Furthermore, no effect was observed on accumulation of MLKL^pS358^ into plasma membrane hotspots by microscopy in HT29 cells (Fig. 8f). In line with their reported mechanisms of action, AT-1001 increased peri-junctional ZO-1 whereas AT-1002 decreases ZO-1 organization (Supplementary Fig. 4g). Unexpectedly, AT-1001 also protected the human leukemia line, Molt-4, from necroptosis (Supplementary Fig. 4f); this was surprising because Molt-4 cells do not express detectable levels of ZO-1 and thus are unlikely to form intercellular tight junctions (Supplementary Fig. 5a). Collectively, these data argue for AT-1001 and AT-1002 serving additional functions as modulators of protein trafficking beyond their reported direct actions upon tight junctions^42^. These observations support the idea that co-regulated trafficking of MLKL and tight junction components underlies the coalescence of MLKL^pS358^ and ZO-1 in epithelial cell plasma membranes.

Reciprocally, our data also indicate that MLKL regulates tight junction formation during necroptosis in epithelial cells. Here, necroptotic stimulation of HT29 cells increased the accumulation of ZO-1 in plasma membrane hotspots in a manner that relied on the expression of MLKL and could be blocked with the MLKL inhibitor, NSA (Fig. 8g, h). Thus, *bona fide* co-regulation exists between MLKL and tight junction proteins during epithelial cell necroptosis. The capacity of tight junction protein trafficking to influence the membranolytic threshold for MLKL represents a new layer of regulation for the necroptotic pathway.

Monocytic U937 cells serve as a negative control for the junctional checkpoints because, compared to epithelial cells, they lack ZO-1 expression (Supplementary Fig. 5a), are refractory to the anti-necroptotic effect of AT-1001 (Supplementary Figs. 4f and  5b, c), and thus exhibit a relatively short temporal gap between the onset of MLKL phosphorylation and death (compare Fig. 1a, b with Supplementary Fig. 5b, c). Furthermore, membrane damage in U937 cells does not manifest as hotspots with ensuing membrane blowout. In stark contrast, necroptotic membrane damage in U937 cells increased in a relatively uniform manner across the plasma membrane, with ensuing death coinciding with the formation of small Annexin V^+^ apertures (Supplementary Fig. 5d, e). Additionally, no facilitation of TNF-induced necroptosis was observed between neighboring U937 cells (Supplementary Fig. 5f). These vastly different features of TNF-induced necroptosis in U937 cells suggest that this cell line is not subjected to the same trafficking and junction-related checkpoints that influence epithelial cell necroptosis.

## Discussion

**Fig. 9:**
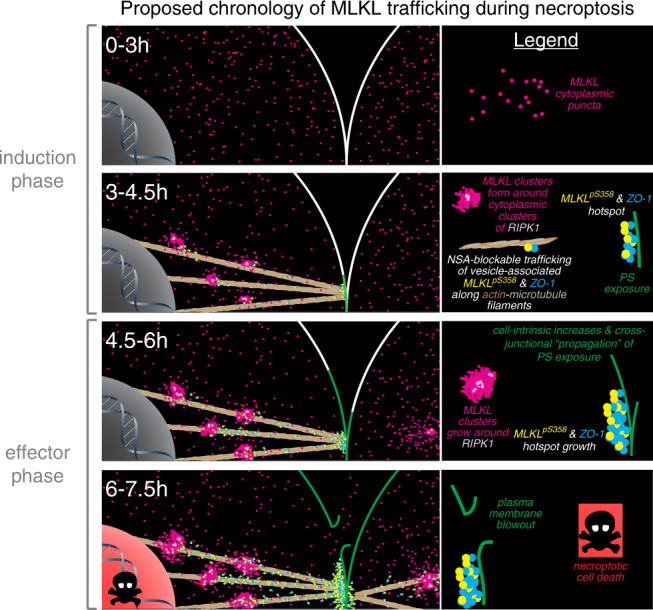
Model of TNF-induced epithelial cell necroptosis chronology. Shown is a graphic summarizing the subcellular relocation and accumulation of RIPK1, MLKL^10C2 or 7G2^, MLKL^pS358^ and ZO-1 that occurs during necroptosis in HT29 cells. The onset and increases in phosphatidylserine (PS) exposure and the eventual onset of plasma membrane blowout are also indicated. Based on the data from this study, a hypothetical explanation for these chronological events during necroptosis is provided.

Here, at the sub-cellular level, we mapped the choreography of activation, trafficking and plasma membrane damage mediated by endogenous human MLKL to execute necroptotic cell death. Based on these data, we propose the following model of epithelial necroptosis (Fig. 9): (1) Under basal conditions, MLKL resides in small (<100 nm) puncta that are distributed evenly throughout the cytoplasm. (2) Between 1.5 and 3 h post-TSI, MLKL phosphorylation, oligomerization and membrane translocation increased as detected by immunoblot analysis. Notably, neither clusters nor hotspots of MLKL are detectable by immunofluorescence at this stage. (3) Between 3 and 4.5 h post-TSI, gel-based analyses show that maximum steady-state levels of MLKL phosphorylation, oligomerization and membrane-association are achieved. MLKL coalesces into large cytoplasmic clusters around congregations of RIPK1, which we propose to be necrosomes. The peri-nuclear location of these clusters, and the observation that Golgi destabilisers attenuated necroptosis, leads us to speculate that clusters form transiently on the surface of the Golgi apparatus. Following this, Golgi-derived vesicles of MLKL^pS358^ emerge from these necrosomal clusters, traffic to the plasma membrane along actin and microtubule filaments, and accumulate with tight junction proteins at nascent hotspots. Concomitantly, damage to the plasma membrane emerges at hotspots during this time. (4) Between 4.5 and 7.5 h post-TSI, clustering at necrosomes and vesicular trafficking to the plasma membrane continues, resulting in a marked increase in the number, size and density of MLKL^pS358^ hotspots. Coordinate plasma membrane damage accumulates at MLKL^pS358^ hotspots until the membranolytic threshold is surpassed, triggering membrane blowout and cell death. As necroptotic hotspots are sites of intercellular junctions in epithelial cells, the accumulation of membrane damage at hotspots has deleterious consequences for adjacent epithelial cells whereby necroptosis of one cell accelerates the death of its abutting neighbors.

Somewhat surprisingly, inhibitors of autophagolysosomal function, nuclear export, clathrin-mediated endocytosis or Cdc42 all altered the extent of cell death when added simultaneously with TSI. Because these inhibitors had negligible effects on the effector phase of necroptosis, it is likely they act upstream or directly upon the necrosome. Consistent with this notion, others have shown that inhibiting autophagolysosomal function enhances RIPK3-mediated MLKL phosphorylation^43^. It has also been shown that inhibiting nuclear export suppresses necroptosis^44^; however, in this instance, nucleocytoplasmic shuttling downstream of MLKL phosphorylation was proposed, which seems incongruous with our finding that Leptomycin B does not influence the effector stage of necroptosis. More broadly, our observations highlight that multiple forms of trafficking regulate the induction phase of necroptosis^45–50^.

By comparison, only the MLKL inhibitor, NSA, or destabilisers of the interrelated microtubule-, actin- and Golgi-trafficking pathways significantly inhibited the effector phase of necroptosis. Because vesicular trafficking of tight junction proteins relies upon these same three pathways to reach the plasma membrane^51^, as a unifying explanation, we propose that MLKL and tight junction proteins converge at the necrosome and thereafter employ the same trafficking mechanism to translocate to the plasma membrane. Several other forms of regulation exist for necroptosis downstream of RIPK3-mediated MLKL phosphorylation^50^. For example, inositol phosphate-6 was proposed to promote necroptosis by mediating pMLKL oligomerization and membrane-association^52,53^; TAM kinases were recently proposed to mediate pMLKL oligomerization^54^; HSP70 and HSP90 likely contribute to pMLKL oligomerization^39,55^; and a trafficking chaperone was proposed to mediate pMLKL membrane translocation via a binding site upon the α4 helix of the human MLKL killer 4HB domain^37^. As the trafficking and junction-related checkpoints described herein influence the effector phase of necroptosis, when pMLKL is already in an oligomeric membrane-associated state, they would likely operate downstream of TAM kinases and inositol phosphate-6 in the necroptotic signaling cascade, potentially in concert with other chaperones.

Internalization and externalization events also regulate necroptosis downstream of the necrosome. For example, Flotillin-mediated endocytosis was proposed to suppress necroptosis by removing MLKL from the plasma membrane and redirecting it for lysosomal degradation^31^. A key role for this degradative pathway, however, was not apparent in our study as Flotillin-1 was largely absent from the plasma membrane during the effector phase of necroptosis, MLKL^pS358^ did not colocalize to a significant extent with LAMP-2-containing lysosomes, and inhibiting lysosomal degradation during the effector phase of necroptosis did not significantly alter the extent of necroptotic cell death. Exocytosis was also proposed to counteract the effector phase of necroptosis via ESCRT-, ALIX:syntenin-1- or Rab27a/b-mediated expulsion of MLKL-containing bubbles to diminish the MLKL residing at the plasma membrane^29–31,53,56^. Here, we observed that Annexin V^+^ membrane bubbles first appeared during the induction phase of necroptosis, then decreased just prior to cell death, consistent with the notion that this phenomenon may allay necroptosis^29^. Nevertheless, our data suggest that membrane bubbling during necroptosis is an incredibly dynamic and heterogenous phenomenon with protrusions variously extending, retracting and shedding in a fashion seemingly independent of the primary sites of MLKL accumulation and membrane damage.

This study provides direct evidence of a mechanism that licenses necroptotic cell death by trafficking MLKL from the necrosome to the plasma membrane. Our data support the idea that a membranolytic threshold must be overcome for MLKL to execute necroptosis. This threshold is met by the accumulation of MLKL into irregularly-shaped hotspots, which is in contrast to highly-ordered protein assemblies, such as Bak/Bax or Gasdermins, which execute other forms of cell death^36^. Remarkably, the post-necrosomal trafficking steps that control the membranolytic threshold also influence the translocation of tight junction proteins during epithelial cell necroptosis. The MLKL hotspots described herein more closely resemble primordial junctions rather than mature epithelial tight junctions^40^. Despite this, epithelial necroptosis has been previously associated with tissue barrier loss; although the basis for this association was not explored^57–60^. Physiologically, our data raise the prospect that necroptotic signaling might be propagated along epithelial tissues, such as the colon, which is consistent with the implication of necroptosis in inflammatory bowel disease^57,58,61^. Moreover, as AT-1001 ameliorates multiple preclinical and clinical examples of gastro-inflammation^41,62,63^ and is undergoing Phase III clinical trials for celiac disease, a role for necroptosis in these indications should be investigated. Because MLKL and tight junction proteins share common trafficking pathways, we propose that the two post-necrosomal checkpoints described herein, (1) trafficking of MLKL via the Golgi/microtubule/actin networks and (2) accumulation of MLKL at plasma membrane hotspots, represent candidates for therapeutic intervention in necroptotic diseases.

## Methods

### Materials

Primary antibodies (and dilution used for immunoblotting) were: rat anti-MLKL (clone 3H1; produced in-house^22^ and available from Merck #MABC604; 1:2000), rat anti-MLKL (clone 7G2; produced in-house; 1:2000), rat anti-MLKL (clone 10C2; produced in-house; 1:2000), rabbit anti-phospho-MLKL phospho-S358 (Abcam #ab187091; 1:1000; referred to as MLKL^pS358^ when used for immunofluorescence), anti-GAPDH (Merck #MAB374; 1:2000), rat anti-human RIPK3 (clone 1H2; produced in-house^11^; 1:2000), rabbit anti-phospho-RIPK3 phospho-S227 (Abcam #ab 209384; 1:1000), rabbit-anti-RIPK1 (Cell Signaling Technology # 3493; 1:1000) mouse anti-Vti1a (BD Biosciences; Cat#6112200), sheep anti-tubulin (Cytoskeleton, Inc. # ATN02), mouse anti-actin (Abcam #ab14128; used in Fig. 7a), anti-β-Actin (Sigma #A1987; used in Supplementary Fig. 2a; 1:2000), anti-VDAC1 (Merck #AB10527; 1:1000), anti-ZO-1 (ThermoFisher Scientific #33-9100; 1:1000), anti-occludin (ThermoFisher Scientific #33-1500; 1:1000), anti-JAM-A (Santa Cruz Biotechnology #sc-53623), anti-TOMM20 (Santa Cruz Biotechnology #sc-17764), anti-LAMP-2 (Santa Cruz Biotechnology #sc-18822), anti-Flotillin (BD Transduction Laboratories #610821), anti-20S (Santa Cruz Biotechnology #sc-374405), anti-HSP90α/β (Santa Cruz Biotechnology #sc-13119), anti-E-cadherin (Santa Cruz Biotechnology #sc-8426), anti-Desmoglein 2 (Santa Cruz Biotechnology #sc-80663). Secondary antibodies for immunoblotting were: donkey anti-mouse IgG (LI-COR Biosciences #925-32212), donkey anti-rabbit IgG (LI-COR Biosciences #925-32213), goat anti-rat IgG (LI-COR Biosciences #925-68029), horseradish peroxidase (HRP)-conjugated goat anti-rat IgG (Southern Biotech #3010-05), HRP-conjugated goat anti-mouse IgG (Southern Biotech #1010-05), and HRP-conjugated goat anti-rabbit IgG (Southern Biotech #4010-05). Secondary immunofluorescence detection reagents were: AlexaFluor647-conjugated donkey anti-rabbit IgG (ThermoFisher Scientific #A31573), AlexaFluor568-conjugated donkey anti-rabbit IgG (ThermoFisher Scientific #A10042), AlexaFluor568-conjugated donkey anti-mouse IgG (ThermoFisher Scientific #A10037), AlexaFluor488-conjugated donkey anti-rat IgG (ThermoFisher Scientific #A21208).

### 10C2 and 7G2 antibody production

Antibodies were generated at the Walter and Eliza Hall Institute Monoclonal Antibody Facility by immunizing Wistar rats with recombinant human MLKL (residues 190-471; expressed and purified from Sf21 insect cells using the baculovirus expression system^24^), before splenocytes were fused with SP2/O mouse myeloid cells and arising hybridoma lines cloned. Specificity of these antibodies for human MLKL was validated via immunoblot analyses as exemplified in Supplementary Fig. 2a, b, and immunofluorescence analyses as exemplified in Supplementary Fig. 2c–e.

*Cell lines –* Wild-type U937 cells were purchased from the American Tissue Culture Collection; HT29 cells were provided by Mark Hampton (University of Otago); HCC2998, SW620, RPMI-8226, Molt-4 and Colo-205 cells from the NCI-60 panel^64^ were provided by Prof. Nick D. Huntington (Walter and Eliza Hall Institute of Medical Research); and YAMC cells were provided by Prof. Robert Ramsay (Peter MacCallum Cancer Centre). The *RIPK1*^*−/−*^, *RIPK3*^*−/−*^ and *MLKL*^*−/−*^ HT29 cells^24,65^ and doxycycline-inducible Mb37-expressing HT29 cells^37^ have been previously reported. To generate the HT29 cells expressing the short MLKL2 splice isoform (as described in ref. ^66^), wild-type and *MLKL*^*−/−*^ HT29 cells were stably transduced with the pF-TRE3G vector (kindly supplied by Dr Toru Okamoto) into which the MLKL2 open reading frame had been introduced to allow doxycycline-inducible expression as described in ref. ^22^. To generate membrane-tagged HT29 and U937 cells, the sequence encoding the mTagRFP-Membrane-1 fusion protein was amplified from Addgene plasmid #57992 using primers: 5’-CAGAATTCATGGCCACCATGCTGTGCTGTATG and 5’- CAGCTAGCTCAATTAAGTTTGTGCCCCAGTTTG. The amplified sequence was subcloned into the pF-TRE3G vector, then transfected into HEK293T cell together with the packaging constructs pCMV∂8.2 and pVSV-G to yield lentivirus. Wild-type HT29 and U937 cells were transduced with this harvested lentivirus and stable expression determined by puromycin selection and flow cytometry.

### Cell culture

HT29 cells were maintained in Dulbecco’s Modified Eagle Medium (DMEM; Life Technologies) containing with 8% v/v heat-inactivated fetal calf serum (FCS), 2 mM L-Glutamine/-GlutaMAX (ThermoFisher Scientific # 35050061), 50U ml^−1^ penicillin and 50U ml^−1^ streptomycin (G/P/S), and HCC2998, SW620, YAMC, RPMI-8226, Molt-4, Colo-205 and U937 cells were maintained in RPMI media containing with 8% v/v FCS and G/P/S under humidified 10% CO_2_ at 37 ^o^C.

### Cell treatment

Cells were seeded into 48-well plates or 8-well μ-Slides (Ibidi) in media containing with 8% v/v FCS and G/P/S at the following density: 3.0 × 10^4^ cells per well for HT29, SW620 or YAMC cultures, 4.0 × 10^4^ cells per well for HCC2998 cultures, and 1.0 × 10^5^ cells per well for U937 cultures. Cells were allowed to equilibrate under humidified 10% CO_2_ at 37 °C conditions (1 h for U937 cells or overnight for all other cell lines) then treated in media containing 1% v/v FCS and G/P/S and supplemented with agonists/antagonists at the following concentrations: 100 ng/mL recombinant human TNF-α-Fc (produced in-house as in ref. ^67^), 500 nM Smac mimetic/Compound A (provided by Tetralogic Pharmaceuticals; as in ref. ^68^) 5 μM IDN-6556 (provided by Idun Pharmaceuticals), 1 μM necrosulfonamide (Merck #480073), 10 μM QVD-OPh (MP Biomedicals #SKU 03OPH10901), 100 μg/mL cycloheximide (Sigma #C4859), 100 ng/mL recombinant human interferon-γ (R&D Systems #285-IF), 400 ng/mL lipopolysaccharide (Sigma #L2630), 1μM ABT-737 (Abbott), 0.1 μM Mcl-1 inhibitor (also known as S63485; custom synthesized by SYNthesis MedChem), 50 μM Nec1s (Merck #504297), 5 μM GSK’872 (SynKinase #SYN-5481), 2 μg/mL Brefeldin A (Sigma #B5936), 50 μM Exo1 (Sigma #E8280), 10 μM Golgicide A (Sigma Cat#G0923), 100 ng/mL Nocodazole (Sigma #M1404), 167 nM Vincristine (Sigma #V8879), 5 μM Chlorpromazine hydrochloride (Sigma #C8138), 30 μM Pitstop 2 (Sigma #SML1169), 100 nM Bafilomycin A (Enzo Life Sciences #BML-CM110-0100), 20.9 μg/mL Latrunculin B (Tocris# 3974), 10 μM Cytochalasin B (Sigma #C6762), 1 μM Leptomycin B (Sigma #L2913), 1 μM ML7 (Sigma #I2764), 100 nM ML141 (Sigma #SML0407), 167 nM MG132 (Merck #474790), 333 μM AT1001 (MedChemExpress #HY-106268A or synthesized to >95% purity by Mimotopes Pty Ltd), 0.417 mg/mL AT1002 (synthesized to >95% purity by Mimotopes Pty Ltd).

### Immunoblots and quantification

Cells were lysed in ice-cold RIPA buffer (10 mM Tris-HCl pH 8.0, 1 mM EGTA, 2 mM MgCl2, 0.5% v/v Triton X100, 0.1% w/v Na deoxycholate, 0.5% w/v SDS and 90 mM NaCl) supplemented with 1x Protease & Phosphatase Inhibitor Cocktail (Cell Signaling Technology #5872S) and 100 U/mL Benzonase (Sigma #E1014). Whole-cell lysates were boiled for 10 min in 1 × SDS Laemmli sample buffer (126 mM Tris-HCl, pH 8, 20% v/v glycerol, 4% w/v SDS, 0.02% w/v bromophenol blue, 5% v/v 2-mercaptoethanol), and resolved by 1.5 mm NuPAGE 4–12% Bis-Tris gel (ThermoFisher Scientific # NP0335BOX) using MES Running buffer (ThermoFisher Scientific # NP000202). After transfer onto polyvinylidene difluoride (Merck, #IPFL00010) membranes were blocked in Odyssey Blocking Buffer (LI-COR #927-50000) then probed with primary antibodies, then the appropriate IR-dye-conjugated secondary antibody and signals revealed with an Odyssey CLx Imaging System (LI-COR). Before the probing with the GAPDH antibody, membranes were incubated in stripping buffer (200 mM glycine pH 2.9, 1% w/v SDS, 0.5 mM TCEP) for 30 min at room temperature then re-blocked. Unless stipulated, immunoblot detection of MLKL was with the rat-anti MLKL (3H1 clone) antibody^22^. Immunoblot signals were exported from ImageStudio Lite software into ImageJ v1.52p for densitometric quantification using the ‘Analyze>Gels’ function.

### Subcellular fractionation

HT29 cells were seeded into 6-well plates (1.0 × 10^6^ cells per well) in media containing with 8% v/v FCS and G/P/S and equilibrated overnight under humidified 10% CO_2_ at 37 °C conditions. Cells were then treated in media containing 1% FCS and G/P/S supplemented with the agonists/antagonists (as indicated above). Cells were fractionated into cytoplasmic and membrane fractions^69^. Cells were permeabilized in MELB buffer (20 mM HEPES pH 7.5, 100 mM KCl, 2.5 mM MgCl_2_ and 100 mM sucrose, 0.025% v/v digitonin, 2 μM N-ethyl maleimide, phosphatase and protease inhibitors). Crude membrane and cytoplasmic fractions were separated by centrifugation (5 min 11,000 × *g*), and fractions prepared in buffers to a final concentration of 1% w/v digitonin. The samples were resolved on a 4–16% Bis-Tris Native PAGE gel (ThermoFisher), transferred to polyvinylidene difluoride (Merck #IPVH00010). After transfer, membranes were destained (in 50% (v/v) Methanol, 25% (v/v) acetic acid), denatured (in 6 M Guanidine hydrochloride, 10 mM Tris pH6.8, 5 mM β-mercaptoethanol), blocked in 5% skim milk (Diploma), probed with primary antibodies then the appropriate HRP-conjugated secondary antibody and signals revealed by enhanced chemiluminescence (Merck #P90720) on a ChemiDoc Touch Imaging System (BioRAD). Between each probe, membranes were incubated in stripping buffer (200 mM glycine pH 2.9, 1% w/v SDS, 0.5 mM TCEP) for 30 min at room temperature then re-blocked.

### LDH release

Colorimetric LDH release assay kit (Promega #G1780) was performed according to manufacturer’s instructions.

### Protein production and purification

Full-length human MLKL and mutants were expressed in Sf21 insect cells using the Bac-to-Bac system (ThermoFisher Scientific) and purified using established procedures^24^. Briefly, proteins were expressed with N-terminal GST tags and captured from lysates using glutathione resin (UBP Bio). Proteins were cleaved on-resin from the GST tag using His-tagged TEV protease, before protease was removed by Ni^2+^ chromatography (HisTag resin, Roche) and protein eluted from a Superdex-200 (GE Healthcare) Size exclusion chromatography column in 200 mM NaCl, 20 mM HEPES pH 7.5, 5% v/v glycerol. Protein containing fractions were spin concentrated to 5–10 mg/mL, aliquoted, snap frozen in liquid N_2_ and stored at –80 °C until required.

### IncuCyte cell death assay

Cells were seeded into 48-well plates and treated (as in *Cell treatment*) in phenol red-free media with 1% FCS, G/P/S, 1 mM Na pyruvate (ThermoFisher Scientific #11360070), 1:500 dilution of AlexaFluor488-conjugated Annexin V (ThermoFisher Scientific #A13201) and 0.5 μg/mL propidium iodide (Sigma #P4170). Cells were moved into an IncuCyte S3 System (Essen Bioscience) and imaged using the 10x objective over time using the default bright-field, green and red channel settings. The number of propidium iodide-positive cells per mm^2^ over time was quantified using IncuCyte S3 v2018A software (Essen Bioscience).

### Immunofluorescence

Cells in 8-well μ-Slides (Ibidi #80826) were ice-chilled for 3 min, then washed in ice-cold Dulbecco’s PBS (dPBS; ThermoFisher Scientific # 14190144), then fixed for 15 min in ice-cold methanol. Cells were washed twice in ice-cold dPBS, then blocked in ice-cold Tris-balanced salt solution with 0.05% v/v Triton-X100 (TBS-T) supplemented with 10% v/v donkey serum (Sigma #D9663) for >1 h. Cells were incubated in primary antibodies (2-4μg/mL anti-MLKL^10C2^ or MLKL^7G2^ antibodies; 1:200 to 1:250 dilution of MLKL^pS358^, ZO-1, occludin or flotillin-1 antibodies; and 1:100 dilution of all other antibodies) overnight at 4 °C in TBS-T with 10% v/v donkey serum. Cells were washed twice in TBS-T then incubated in the appropriate secondary antibodies supplemented with 0.1–0.5 μg/mL Hoechst 33342 (ThermoFisher Scientific #H3570) for 3 h at room temperature with gentle rocking. Cells were washed four times in ice-cold TBS-T then stored at 4 °C until being imaged. Where indicated, to demarcate the plasma membrane, 2μL of biotinylated wheat germ agglutinin (Sigma #L5142) was added to each well 10 min before fixation, and fixed wheat germ agglutinin was then detected via the addition of 1–2 μg/mL DyLight650-conjugated streptavidin (ThermoFisher Scientific #84547) during the secondary antibody incubation step. Importantly, as ~ 95% of propidium iodide-positive cells are lost during this immunofluorescence protocol, it provides insight into events prior to necroptotic cell death.

### 2-dimensional epifluorescence microscopy

Samples in TBS-T were imaged on an Inverted Axio Observer.Z1 microscope (Zeiss) with the following specifications: C-Apochromat 40x/1.20 W autocorr UV VIS IR lens, HXP 120 V excitation source, AlexaFluor647 and DyLight650 imaged with a λ_Excitation_ = 625–655 nm; λ_beamsplitter_ = 660 nm; λ_Emission_ = 665–715 nm filter, AlexaFluor568 imaged with a λ_Excitation_ = 532–544 nm; λ_beamsplitter_ = 560 nm; λ_Emission_ = 573–613 nm, AlexaFluor488 imaged with a λ_Excitation_ = 450–490 nm; λ_beamsplitter_ = 495 nm; λ_Emission_ = 500–550 nm, Hoechst 33342 imaged with a λ_Excitation_ = 359–371 nm; λ_beamsplitter_ = 395 nm; λ_Emission_ = 397–∞ nm, a sCMOS PCO.edge 4.2 camera, ZEN blue 2.5 pro capture software and ImageJ 1.52p post-acquisition processing software^70^. Typically, for each independent experiment, 10–20 randomly selected fields were captured per treatment group, whereby only the Hoechst signal was visualized prior to multi-channel acquisition. To ensure consistent signal intensities across independent experiments, the same excitation, emission and camera settings were used throughout this study.

### Structured illumination or deconvolved wide field microscopy

Fixed immunostained cells in 8-well μ-Slides (Ibidi #80827) were subjected to super-resolution 3-dimensional structured illumination microscopy on a DeltaVision OMX-SR system (GE Healthcare) equipped with a 60x/1.42 N.A. PlanApo oil immersion objective (Olympus), sCMOS cameras, and 405-, 488-, 568- and 640-nm lasers, and 1.520 refractive index immersion oil. Image stacks were acquired consisting of 15 raw images per plane (5 phases, 3 angles) per color channel and a z-step size of 125 nm. Raw images for widefield deconvolution were acquired at one image per plane per color channel and a z-step size of 375 nm. Super-resolution reconstruction or widefield deconvolution and color channel alignment were performed with softWoRx v7.0 (GE Healthcare).

### Lattice Light Sheet Microscopy (LLSM)

Cells were seeded onto glass coverslips in phenol red-free media with 1% FCS, G/P/S, 1 mM Na pyruvate (ThermoFisher Scientific #11360070), 1-in-500 dilution of AlexaFluor488-conjugated Annexin V (ThermoFisher Scientific #A A23204) and 0.1μΜ TO-PRO-3 iodide (ThermoFisher Scientific #T3605). Cells were moved onto a custom-built LLSM system based on prior design^71^, treated with TSI (as in *Cell treatment*) and incubated under humidified 10% CO_2_ at 37 °C. Samples were illuminated using 488-, 560-, and 641-nm diode lasers (MPB Communications) through an excitation objective (Special Optics, 0.65 NA). The LLSM was illuminated at the back aperture of the excitation objective through an annular mask of 0.325 inner NA and 0.4 outer NA providing a light sheet length of 20 microns. Fluorescence was collected by a detection objective (Nikon, CFI Apo 25X, 1.1 NA) and by a sCMOS cameras (Hamamatsu Orca Flash 4.0 v2). Volumes of 100 × 72 × 20 microns were acquired at a frame speed of 20 milliseconds giving a volume time of 15 sec with a time interval of 2 min between volumes. Acquired data were de-skewed then deconvolved using an iterative Richardson-Lucy algorithm. Data was compressed to h5 format using the BigDataViewer plugin^72^ in ImageJ 1.52p^70^ for visualization and analysis in Imaris v8-9 (Bitplane).

### Confocal microscopy

Cells were seeded 8-well μ-slides (Ibidi #80826) and TSI-treated (as in *Cell treatment*) in phenol red-free media with 1% FCS, G/P/S, 1 mM Na pyruvate (ThermoFisher Scientific #11360070), 1-in-500 dilution of AlexaFluor488-conjugated Annexin V (ThermoFisher Scientific #A13201) and 0.5 μg/mL propidium iodide (Sigma #P4170). Cells were moved onto an Inverted LSM 880 confocal microscope (Zeiss) with the following specifications: Plan-Apochromat 20×/0.80 air lens, 405-, 488-, 561-, 633-nm laser lines, Zeiss photomultiplier tube, ZEN black 2.3 SP1 FP3 v14.0 capture software and ImageJ 1.52p post-acquisition processing software^70^. Cells were maintained under humidified 5% CO_2_ at 37 °C and imaged every 15 min for 7.5 h. Cells were then fixed, immunostained (as in *Immunofluorescence*) and re-imaged with comparable confocal settings.

### Airyscan microscopy

Fixed immunostained cells in 8-well μ-Slides (Ibidi #80827) were subjected to super-resolution 3-dimensional Airyscan microscopy on an Inverted LSM 880 platform (Zeiss) equipped with the following specifications: a 63x/1.4 N.A. PlanApo DIC M27 oil immersion objective (Zeiss), 405-, 488-, 568- and 640-nm laser lines, and radially-stacked Airyscan GaASP detectors set to SR-mode, 405-, 488-, 561-, 633-nm laser lines and ZEN black 2.3 SP1 FP3 v14.0 capture software. Image stacks were acquired with a z-step size of 159 nm. Super-resolution deconvolution was performed using the automated ‘3D AiryScan Processing’ function of ZEN blue software.

### Colocalization quantitation

Wild-type HT29 cells were TSI-treated for 7.5 h, fixed and immunostained with the MLKL^10C2^, ZO-1 and MLKL^pS358^ antibodies (as in *Immunofluorescence*). Six randomly selected micrographs from *n* = 2 independent experiments were used to gauge the degree of signal co-proportionality between MLKL^10C2^, ZO1 and MLKL^pS358^ immunosignals at the single-pixel level. This approach is based upon the recommendations of^73^ and carries a *x,y*-resolution limit of ~ 350 nm. In brief, a rolling ball filter of 10 was applied to all channels and thresholded areas from all channels were processed using the ‘Process>Image calculator>add’ tool to create a universal mask. This mask removes low and non-specific signals from the analysis. This mask was then processed using the Coloc 2 plugin in ImageJ 1.52p^70^. The 2-dimensional histograms generated by the Coloc 2 plugin plotting the ‘MLKL^10C2^ versus MLKL^pS358^’ and the ‘ZO-1 versus MLKL^pS358^’ immunosignals were then averaged as shown in Supplementary Fig. 2g or Fig. 8b, respectively. Pearson’s correlation coefficient of determination (R^2^-value) generated by the Coloc 2 plugin was used to further gauge the degree of colocalization. 6 fields where the MLKL^pS358^ immunosignal was randomly redistributed were used as additional non-correlating controls for Pearson’s analysis.

### Hotspot quantitation

Micrographs (captured as in *2-dimensional epifluorescence microscopy*) of the MLKL^pS358^ immunofluorescent signal were opened in ImageJ 1.52p^70^. A rolling ball filter of 5 was applied, MLKL ^pS358^ immunosignals thresholded (≥9000 units) and objects segmented using the ‘Analyze>Analyze Particles’ tool. Segmented objects with size 0.5–100 μm^2^ and feret diameter>2 (i.e., elliptical objects) were considered hotspots. The number of segmented objects per 100 cells was taken as an index of hotspot occurrence. The mean size of the segmented objects was taken as an index of hotspot size. The fluorescence intensity of each segmented objected divided by its size was taken as an index of hotspot intensity. Quantitation of ZO-1 hotspots was performed using the same method, but with a rolling ball filter of 7 and thresholded immunosignals ≥7000 units were measured.

### MLKL cluster quantitation

Micrographs (captured as in 2-dimensional epifluorescence microscopy) of the MLKL^7G2^ immunofluorescent signal were opened in ImageJ 1.52p^70^. A rolling ball filter of 6 was applied, MLKL^7G2^ immunosignals thresholded (≥2000 units) and objects segmented using the ‘Analyze>Analyze Particles’ tool. Segmented objects with size 0.5–50 μm^2^ and circularity 0.6-1 were considered clusters. The number of segmented objects in TSI-treated *MLKL-/-* HT29 cells (i.e., an index of non-specific objects) was subtracted from the number of segmented objects in wild-type HT29 cells. The remaining number of segmented objects per 100 cells was taken an index of cluster occurrence. The mean size of the segmented objects was taken as an index of cluster size. The fluorescence intensity of each segmented object divided by its size was taken as an index of cluster intensity. This method of MLKL^7G2^-positive clusters was used for Fig. 2d and Supplementary Fig. S2h. A similar method was used to quantify MLKL^10C2^-positive clusters in Fig. 7c and Supplementary Fig. 3f, except that a rolling ball filter of 7 was applied and thresholded immunosignals ≥5000 units were measured.

### Quantitation of spatiotemporal gaps between death events

The ‘red channel’ micrographs of propidium iodide-uptake over time from the aforementioned Incucyte experiments (as in *Incucyte cell death assay* with frames taken every 5 min) were exported into ImageJ 1.52p^70^. A rolling ball filter of 50 was applied, registration was performed using the StackReg plugin^74^, and the spatial and temporal coordinates of propidium iodide-positive cells tracked over time using the Trackmate plugin^75^. The result.txt file of coordinates was parsed using a custom-designed visual basic macro to determine the spatial and temporal gap between cell death events for all propidium iodide-positive cells in the field. One cell width was approximated to be 40 pixels and so spatial gaps of ≤40, 40–80 and 80–120 pixels were binned into 1, 2 and 3 units of cell width, respectively.

### Statistical analyses

The number of independent experiments for each dataset is stipulated in the respective figure legend. The employed statistical analyses are stipulated in the respective figure legend and performed using Prism v.8.2 (GraphPad). Statistical analyses were only performed on datasets collated from at least three independent experiments.

### Reporting summary

Further information on research design is available in the Nature Research Reporting Summary linked to this article.

## Supplementary information

Supplementary Information

Peer Review

Reporting Summary

Description of Additional Supplementary Files

Supplementary Movie 1

Supplementary Movie 2

Supplementary Movie 3

Supplementary Movie 4

## Data Availability

All data and customised Image J macros used by study are available on request from the authors. The source data underlying Figs. 1a–c, 3b, d, e, 4c, 5c, 6b–d, 8b–e, g, and Supplementary Figs. 1a–f, 2a, b, g, h, 3a–f, 4d–f, 5a–c, e, f are provided as a Source Data file. Source data are provided with this paper.

## Source data

Source Data
