# Supplementary material for: MLKL trafficking and accumulation at the plasma membrane control the kinetics and threshold for necroptosis
Source: Nat Commun. 2020 Jun 19;11:3151. doi: 10.1038/s41467-020-16887-1 (PMC7305196; doi:10.1038/s41467-020-16887-1)
Supplement: Supplementary file 1 — Supplementary Information [file 41467_2020_16887_MOESM1_ESM.pdf]

SUPPLEMENTARY INFORMATION

MLKL trafficking and accumulation at the plasma membrane control the kinetics and threshold for necroptosis

Samson *et al.*

Supplementary Fig. 1. Multiple epithelial cell lines exhibit a temporal gap between MLKL phosphorylation and necroptotic plasma membrane lysis. HT29 (**a,b**), HCC2998 (**c,d**), SW620 (**e,f**) cells were treated with media alone or the indicated necroptotic stimuli in the presence/absence of the MLKL-selective inhibitor, necrosulfonamide (NSA). The degree of MLKL phosphorylation and plasma membrane lysis over time was then measured. (**a,c,e)** Whole cell lysates were subjected to SDS-PAGE and immunoblotted to determine the extent of MLKL phosphorylation. (**b,d,f)** Left-hand *y*-axes plot the ratio of the pMLKL and MLKL immunoblot signals from (**a,c,e)** and are representative of data from n=2-3 independent experiments per cell line. Right-hand *y*-axes plot the extracellular release of LDH (relative to detergent lysed cells) as an index of plasma membrane lysis from the same experiment, and representative of data from n=2 (for TSQ-treated HT29 cells) and n=3 (for TSI-treated HCC2998 and SW620 cells) independent experiments. Mean ± s.e.m of N=4 biological replicates.

Supplementary Figure 1

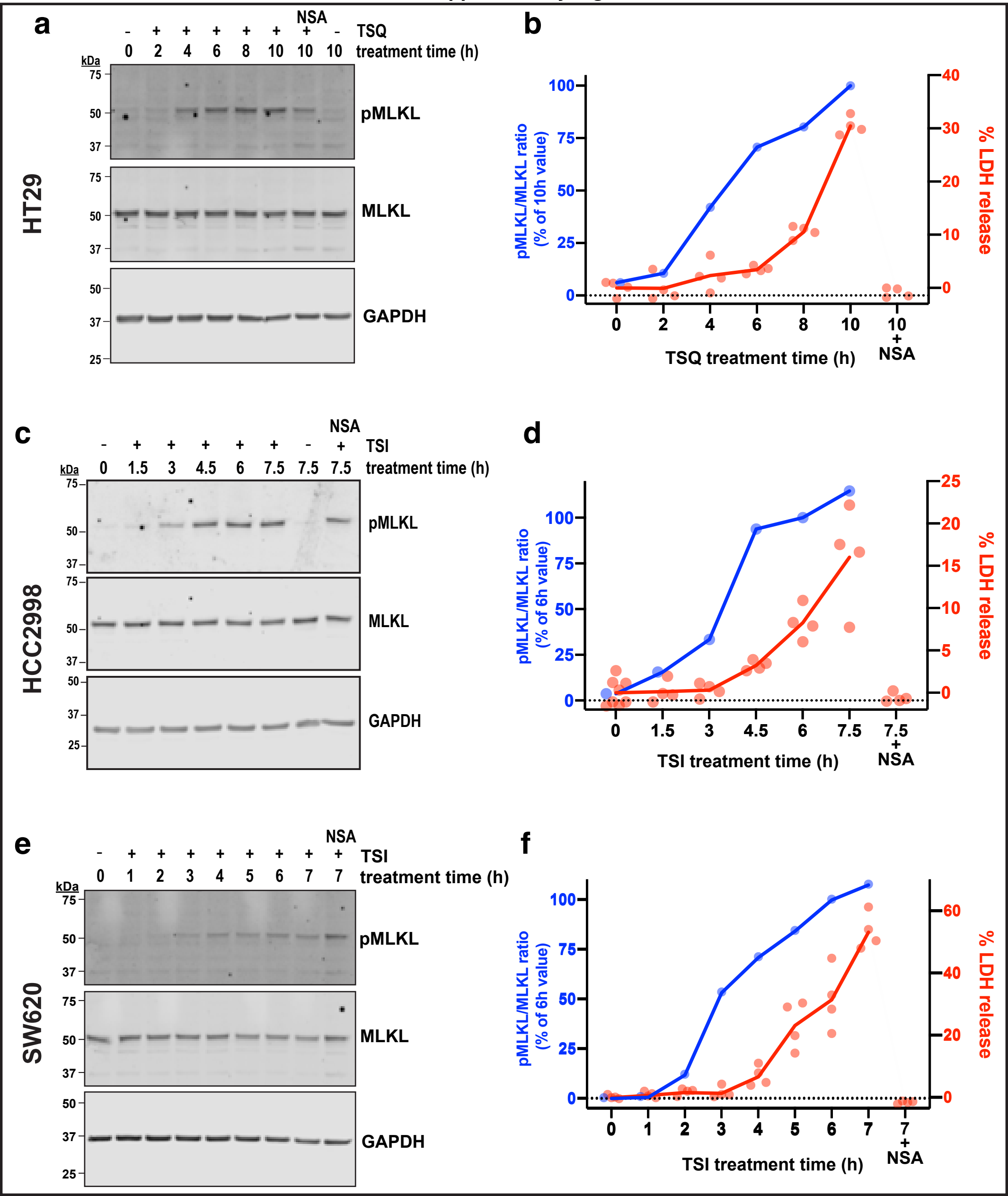

Supplementary Fig. 2 Immunofluorescent detection of different stages of MLKL activation. (a) Wild-type and *MLKL*$^{-/-}$ HT29 cells were treated overnight ±20ng/mL doxycycline to induce expression of the short MLKL2 isoform of human MLKL (MLKL^{short})[1] and then TSI-treated for 7.5 hours. Whole cell lysates were fractioned by SDS-PAGE and immunoblotted for the MLKL-specific antibodies: the MLKL^{pS358} antibody[2], 3H1[3], 10C2 and 7G2 (this work). As the 10C2 antibody recognises both the full-length and the short MLKL2 isoform (which contains only a C-terminal section of the pseudokinase domain), the linear epitope maps to between residues G414-K471 in human MLKL. **(b)** 1μg of recombinant full-length human MLKL (WT) and three mutated variants (C86S, F386A and T357E/S358E) were fractioned by SDS-PAGE and immunoblotted to show that the anti-MLKL 7G2 clone antibody specifically recognises a F386-containing linear epitope. **(c)** HT29 cells were stimulated with the indicated necroptotic stimuli: TSI for 7.5 hours, TSQ (TNF/Smac mimetic/QVD-OPh) for 10 hours, TCI (TNF/cycloheximide/IDN-6556) for 7.5 hours, ISI (Interferon-γ/Smac mimetic/IDN-6556) for 12 hours and LSI (Lipopolysaccharide/Smac mimetic/IDN-6556) for 13 hours. Cells were then immunostained for MLKL^{10C2} and MLKL^{pS358}, DNA counterstained with Hoechst, then imaged via epifluorescence and differential interference contrast (DIC) microscopy. Clusters and hotspots were observed during TNF-induced necroptosis. Hotspots, but not clusters, were observed during Interferon- or Lipopolysaccharide-induced necroptosis. **(d)** Wild-type, *RIPK1*$^{-/-}$, *RIPK3*$^{-/-}$ or *MLKL*$^{-/-}$ HT29 cells were TSI-treated for 7.5 hours then immunostained (as in **c**). **(e)** HT29 cells were treated for 7.5 hours with TSI to induce necroptosis, TS to induce extrinsic apoptosis or ABT737+Mcl1 inhibitor (A+M) to induce intrinsic apoptosis, then immunostained for MLKL^{7G2} and MLKL^{pS358}. Micrographs in **(c-e)** are representative of n=2-8 independent experiments. **(f)** HCC2998 were TSI-treated for 7.5 hours then immunostained (as in **d**). Micrographs are representative of n=3 experiments. **(g)** HT29 cells were TSI-treated for 7.5 hours, immunostained and imaged (as in **c** with a *x,y*-resolution of ~350nm). Scatterplot depicts the MLKL^{10C2} and MLKL^{pS358} pixel co-intensities across N=514 cells from n=2 independent experiments. Negligible proportionality was observed between the MLKL^{10C2} and MLKL^{pS358} immunosignals. **(h)** Left axes plot the frequency, intensity and size of individual clusters and hotspots during TSI-induced necroptosis of HT29 cells from one experiment (N=1258-1973 cells per timepoint) and representative of n=3 independent experiments. Population means are indicated by lines. **Fig. 2d** is a multiparametric version of the same data. Right axes plot the total MLKL^{10C2} and MLKL^{pS358} immunosignal intensity per cell.

Supplementary Figure 2

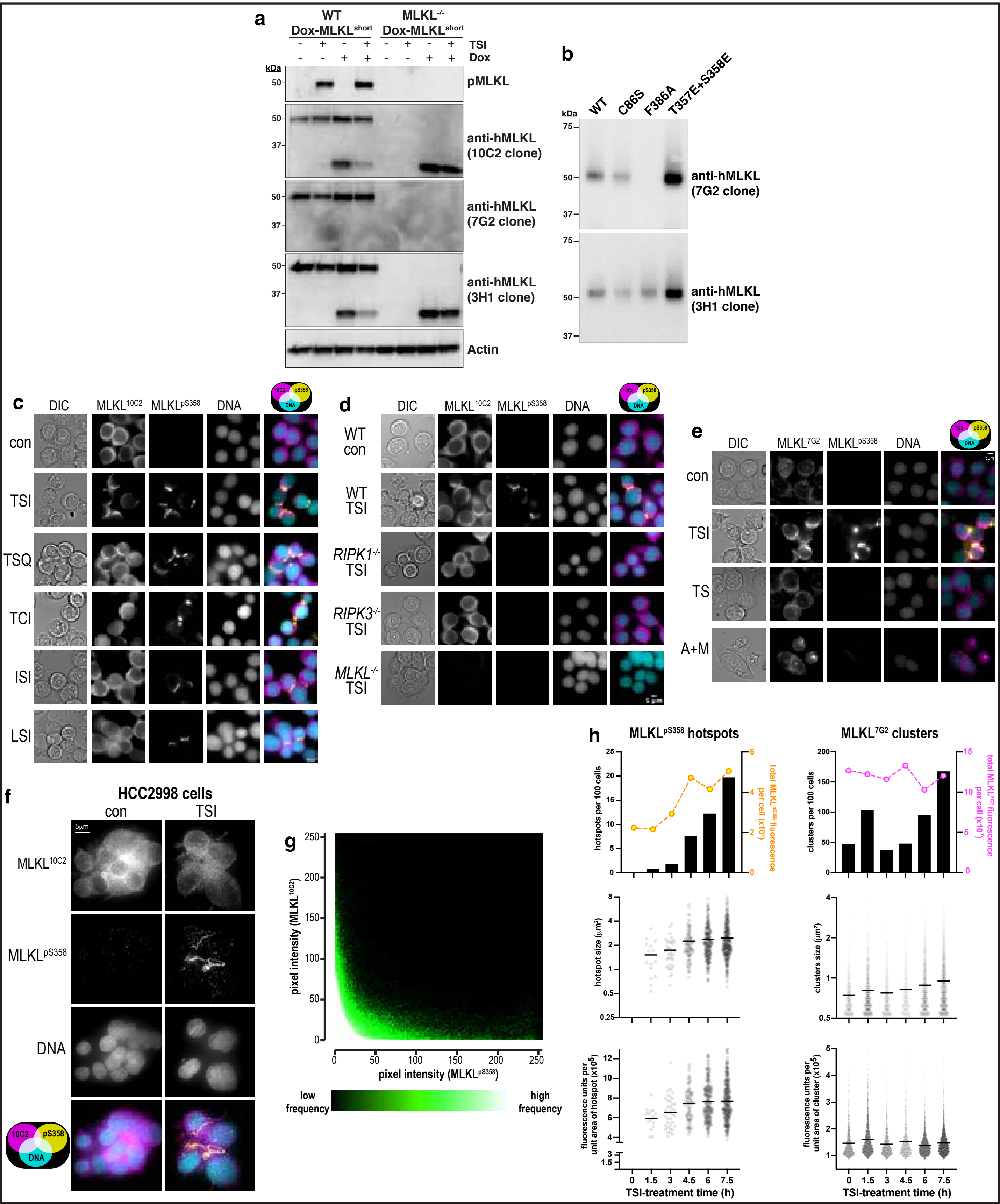

Supplementary Fig. 3. Inhibiting microtubule-, actin- and Golgi-based trafficking slows necroptosis in many, but not all, cell types. (a) The indicated inhibitors were added to HT29 cells 0, 5 or 6 hours after TSI-treatment. Extracellular LDH release (relative to detergent-treated cells) was measured after 7.5 hours of TSI-treatment as an index of plasma membrane lysis. Each red dot represents the mean of N=3-4 biological replicates from one independent experiment. Bars indicate mean + s.e.m. across n=3-4 independent experiments. **Fig. 4b** summarizes the same data. **(b)** Mouse YAMC epithelial cells were treated with TSI ±NCB. Extracellular LDH release (relative to detergent-treated cells) was measured after 1.5 hours of TSI-treatment as an index of plasma membrane lysis. Each red dot represents the mean of N=3 biological replicates from one independent experiment. Bars indicate mean + SEM across n=4 independent experiments. *p=0.0112 via paired two-tailed t-test. **(c)** NCB was added after 5 hours treatment of HT29 cells with TSI (top), TS (middle) and ABT737/Mcl-1 inhibitor (lower). The number of propidium iodide $(PI)^+$ (dead) and Annexin V^+ cells per mm^2 over time were assessed using an IncuCyte S3 System. Data are mean ± s.e.m. of N=3 fields with >600 cells per group from n=1 independent experiment. Data are representative of n=2 independent experiments. **(d)** HT29 cells were treated with NCB 0, 5 or 6 hours post-TSI. After 7.5 hours of TSI-treatment, **s**ubcellular fractions of the cytosol (C) and membrane (M) were prepared, subjected to Blue Native-PAGE and immunoblotted to assess MLKL phosphorylation, oligomerization and membrane-association. Fractionation was verified by probing for the membrane protein, VDAC1, and the cytosolic protein, GAPDH. Data are from n=1 independent experiment. **(e)** Cells were treated with TSI in the ±NSA or NCB and the number of propidium iodide $(PI)^+$ (dead) cells over time was assessed using an IncuCyte S3 System. The number of dead cells in the presence of NSA or NCB was measured after 5 hours (for U937, RPMI-8226, Colo-205) or 10 hours (for SW620 or Molt-4) of TSI-treatment. Data are expressed relative to the number of dead cells after TSI-treatment alone (as an index of decreases in the rate of necroptotic cell death). Bars indicate mean + s.e.m. across n=1-4 independent experiments. Each red dot represents the mean of N=3-4 biological replicates from one independent experiment. **(f)** NCB was added to HT29 cells after 5 hours of TSI-treatment. After 7.5 hours of treatment, cells were immunostained for $MLKL^{10C2}$ and $MLKL^{pS358}$. DNA was counterstained with Hoechst and cells imaged via epifluorescence microscopy. Each dot represents the mean frequency, intensity and size of clusters and hotspots from one independent experiment (N=2830 and 2181 cells for the TSI and TSI+NCB@5h groups, respectively).

Supplementary Figure 3

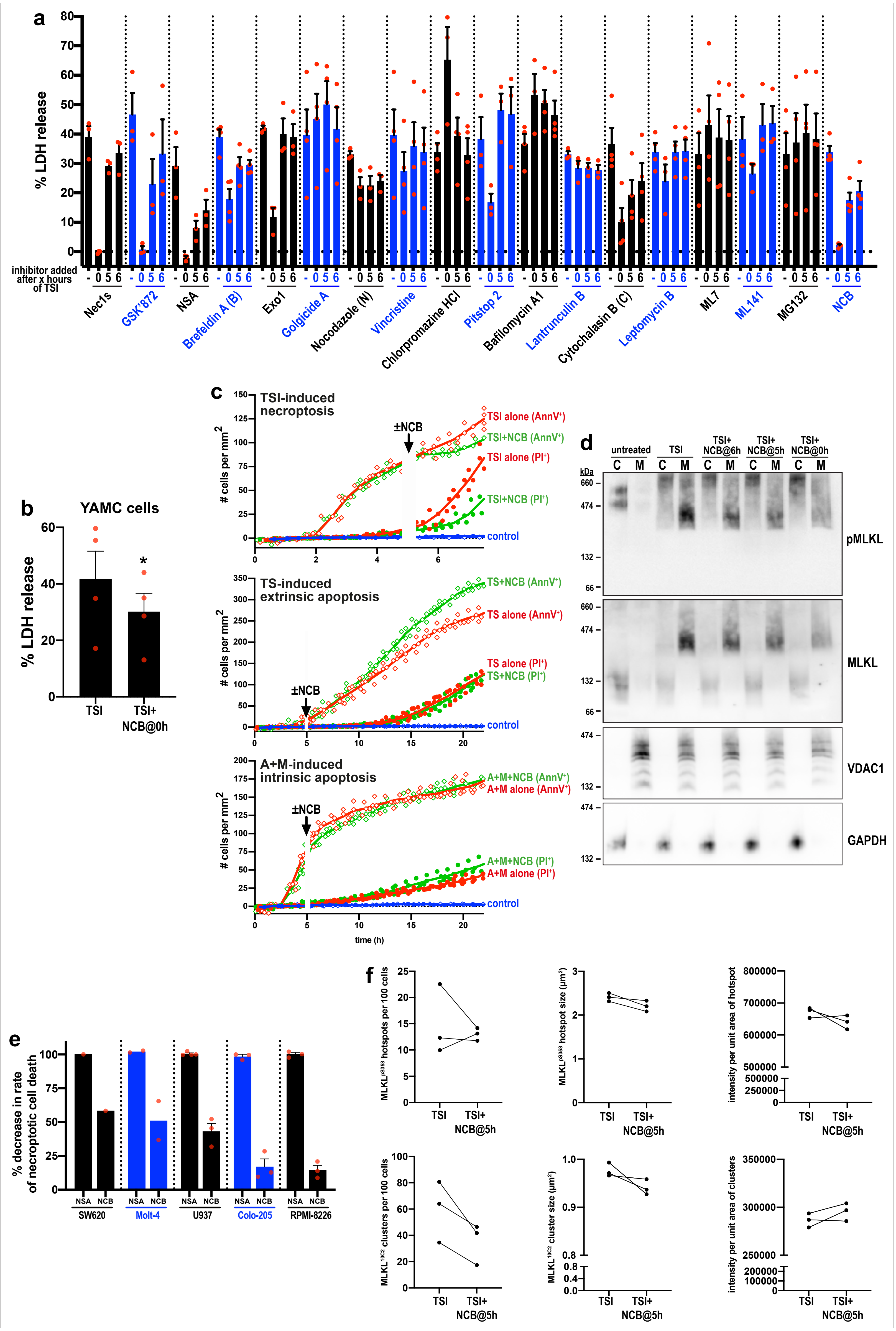

Supplementary Fig. 4. MLKL and tight junction protein trafficking is co-regulated during necroptosis. (a,b) HT29 cells were left untreated (control) or TSI-treated for 7.5 hours then subjected to immunofluorescence for $MLKL^{7G2}$ (**a**) and $MLKL^{10C2}$ (**b**), $MLKL^{pS358}$ and the indicated markers. Cells were imaged via epifluorescence and differential interference contrast (DIC) microscopy. Flotillin-1 resides at both plasma and internal membranes in untreated cells. However, after 7.5 hours of TSI-treatment Flotillin-1 does not reside at the plasma membrane and thus it does not colocalize with the $MLKL^{pS358}$ hotspots (arrows). LAMP-2, 20S proteasome, TOMM20, and HSP90, E-cadherin and Desmoglein-2 also exhibit negligible colocalization with pMLKL hotspots. The tight junction proteins, ZO-1, JAM-A and Occludin, all exhibit a high degree of colocalization with $MLKL^{pS358}$ hotspots. Micrographs are representative of n=2-10 independent experiments. (**c**) HCC2998 cells were left untreated (control) or TSI-treated for 7.5 hours then subjected to immunofluorescence for $MLKL^{10C2}$, $MLKL^{pS358}$ and ZO-1. DNA was counterstained with Hoechst and cells imaged via deconvolved 3-dimenstional widefield microscopy. Micrographs are a maximum intensity projection and representative of n=3 independent experiments. **(d,e)** Human HCC2998 (**d**) and mouse YAMC (**e**) epithelial cells were treated with TSI in the presence or absence of AT-1001 and extracellular LDH release was measured as an index of plasma membrane lysis after 7.5 hours (**d**) or 1.5 hours (**e**) of treatment. Bars indicate mean + SEM across n=2 (for HCC2998 cells) and n=4 (for YAMC cells) independent experiments. Dots represent the mean of N=3-4 biological replicates from each independent experiment. *p=0.0245 via paired two-tailed t-test for the anti-necroptotic effect of AT-1001. **(f)** Cells were treated with TSI ±NSA or AT-1001 and the number of propidium iodide $(PI)^{+}$ (dead) cells over time was assessed using an IncuCyte S3 System. The data are expressed as in **Supplementary Fig. 3e**. Bars indicate mean + s.e.m. across n=1-4 independent experiments. Each red dot represents the mean of N=3-4 biological replicates from one independent experiment. The same data showing inhibition of necroptosis by NSA appears in **Supplementary Fig. 3e**. **(g)** Wild-type HT29 cells were treated with for 7.5 hours with TSI with/without AT1001 or AT1002 then immunostained for ZO-1 and imaged via epifluorescence microscopy. Micrographs are representative of N=803-1458 cells per group from one independent experiment.

Supplementary Figure 4

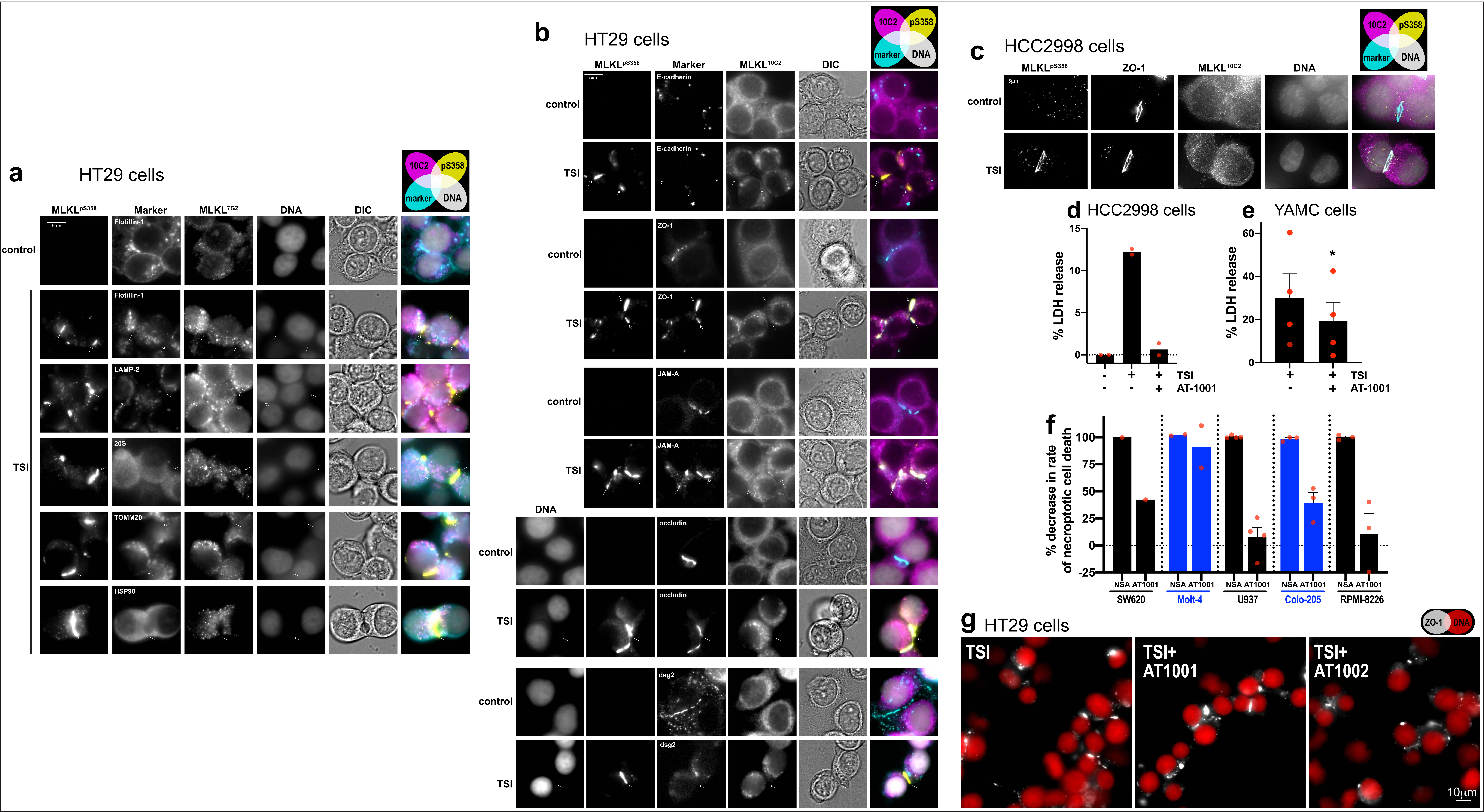

Supplementary Fig. 5. The necroptotic effector phase of U937 cells is regulated differently to that of epithelial cells. (a) Whole cell lysates of untreated wild-type Colo-205, U937, HCC2998, SW620, HT29 and *MLKL*$^{-/-}$ HT29 cells were fractioned by SDS-PAGE and immunoblotted to determine their relative expression of ZO-1 and MLKL. Data are representative of n=2 independent experiments. **(b,c)** U937 cells were TSI-treated ±NSA, NCB or AT1001. The degree of MLKL phosphorylation (**b**) and cell death (**c**) over time was then measured. **(b)** Whole cell lysates were subjected to SDS-PAGE and immunoblotted to determine the extent of MLKL phosphorylation. **(c)** Left-hand *y*-axes plot the ratio of the pMLKL and MLKL immunoblot signals from **b** and representative of data from n=2 independent experiments. Right-hand *y*-axes plot the number of propidium iodide (PI)$^+$ (dead) cells per mm^2 over time were assessed via an IncuCyte S3 System from n=1-4 independent experiments. Mean ± s.e.m of N=4 biological replicates. **(d,e)** U937 cells that constitutively express mTagRFP-Membrane-1 fusion protein were TSI-treated. Annexin V binding and TOPRO-uptake were then imaged via time-lapse LLSM. Micrographs are maximum intensity projections and representative of N=30-41 cells across n=3 independent experiments. The treatment time for each micrograph is shown. 3D micrographs of **d** are shown in **Supplementary Video 3**. **(e)** Cells which shed or retained bubbles preceding death, which formed hotspots preceding death, and which featured membrane blowout or pore-like structures contemporaneously with death were counted (expressed as a percentage of all cells; N=30-41 per group across n=3 independent experiments). **(f)** U937 cells were treated with TSI then the onset of cell death (i.e. propidium iodide-uptake) and subsequent movement of dead cells was tracked over time using the Incucyte S3 System. Graph shows the temporal gap and spatial gap (binned into cell width units) between death events across the whole population. Each dot represents the spatiotemporal gap between two dead cell events. Mean ± 95% confidence intervals are shown and the dashed line represents the mean spatiotemporal gap between all cell death events across the population. No significant differences ($p>0.05$) between the groups and the population was detected via one-way ANOVA with Tukey's correction for multiple comparisons. N=71 gaps for 1-cell-width group, N=230 for 2-cell-width group, N=363 for 3-cell-width group and N=21831 gaps for the whole population.

Supplementary Figure 5

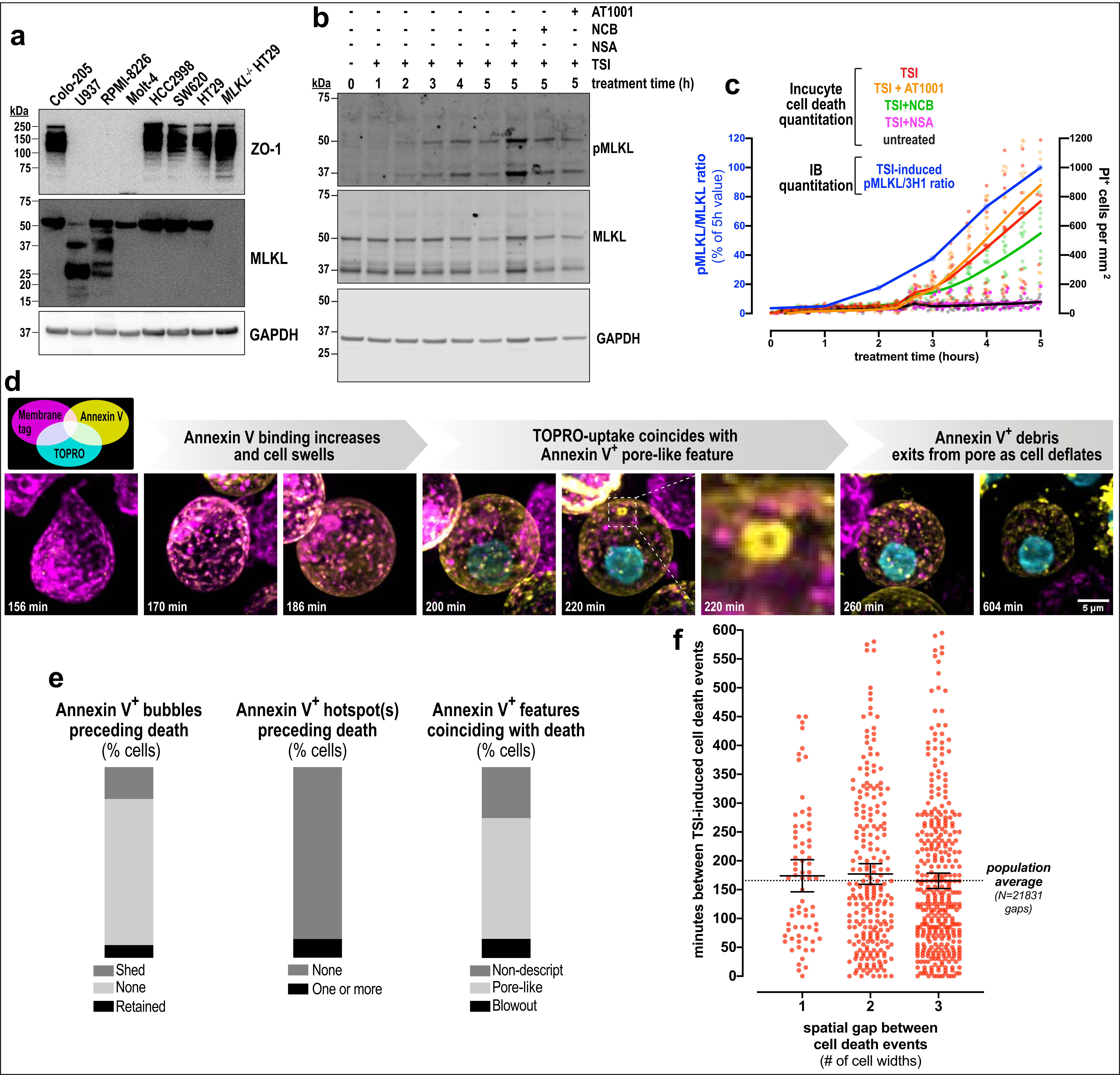

Supplemental References

1. Arnez, K.H. *et al.* Analysis of the N-terminal region of human MLKL, as well as two distinct MLKL isoforms, reveals new insights into necroptotic cell death. *Biosci Rep* **36**, e00291 (2015).
2. Wang, H. *et al.* Mixed lineage kinase domain-like protein MLKL causes necrotic membrane disruption upon phosphorylation by RIP3. *Mol Cell* **54**, 133-146 (2014).
3. Murphy, J.M. *et al.* The pseudokinase MLKL mediates necroptosis via a molecular switch mechanism. *Immunity* **39**, 443-453 (2013).
