# Supplementary material for: MLKL trafficking and accumulation at the plasma membrane control the kinetics and threshold for necroptosis
Source: Nat Commun. 2020 Jun 19;11:3151. doi: 10.1038/s41467-020-16887-1 (PMC7305196; doi:10.1038/s41467-020-16887-1)
Supplement: Supplementary file 3 — Reporting Summary [file 41467_2020_16887_MOESM3_ESM.pdf]

nature research

Corresponding author(s): Andre Samson, Edwin Hawkins & James Murphy

Last updated by author(s): May 25, 2020

Reporting Summary

Nature Research wishes to improve the reproducibility of the work that we publish. This form provides structure for consistency and transparency in reporting. For further information on Nature Research policies, see Authors & Referees and the Editorial Policy Checklist .

Statistics

For all statistical analyses, confirm that the following items are present in the figure legend, table legend, main text, or Methods section.

n/a	Confirmed	
☐	☒	The exact sample size (*n*) for each experimental group/condition, given as a discrete number and unit of measurement
☐	☒	A statement on whether measurements were taken from distinct samples or whether the same sample was measured repeatedly
☐	☒	The statistical test(s) used AND whether they are one- or two-sided<br>*Only common tests should be described solely by name; describe more complex techniques in the Methods section.*
☐	☒	A description of all covariates tested
☐	☒	A description of any assumptions or corrections, such as tests of normality and adjustment for multiple comparisons
☐	☒	A full description of the statistical parameters including central tendency (e.g. means) or other basic estimates (e.g. regression coefficient) AND variation (e.g. standard deviation) or associated estimates of uncertainty (e.g. confidence intervals)
☐	☒	For null hypothesis testing, the test statistic (e.g. *F*, *t*, *r*) with confidence intervals, effect sizes, degrees of freedom and *P* value noted<br>*Give P values as exact values whenever suitable.*
☒	☐	For Bayesian analysis, information on the choice of priors and Markov chain Monte Carlo settings
☒	☐	For hierarchical and complex designs, identification of the appropriate level for tests and full reporting of outcomes
☐	☒	Estimates of effect sizes (e.g. Cohen's *d*, Pearson's *r*), indicating how they were calculated

Our web collection on statistics for biologists contains articles on many of the points above.

Software and code

Policy information about availability of computer code

Data collection	The equipment and methods used to acquire data are fully disclosed in the manuscript. If required, more information (e.g. model numbers of equipment) can also be provided.
Data analysis	The softwares used to analyze data were Excel for Mac v16.35 (Microsoft), Prism v.8.2 (GraphPad), ImageJ 1.52p (NIH), Imaris v8-9 (Bitplane) and ZEN black 2.3 (Zeiss). The methods used to analyze data are fully disclosed in the manuscript. If required, more information (e.g. the code for the Image J macros) can also be provided.

For manuscripts utilizing custom algorithms or software that are central to the research but not yet described in published literature, software must be made available to editors/reviewers. We strongly encourage code deposition in a community repository (e.g. GitHub). See the Nature Research guidelines for submitting code & software for further information.

Data

Policy information about availability of data

All manuscripts must include a data availability statement. This statement should provide the following information, where applicable:

- Accession codes, unique identifiers, or web links for publicly available datasets
- A list of figures that have associated raw data
- A description of any restrictions on data availability

All data and customised Image J macros used by study are available on request from the authors. The source data underlying Figures 1a-c, 3b, 3d-e, 4c, 5c, 6b-d, 8b-e, 8g, and Supplementary Figures 1a-f, 2a-b, 2g-h, 3a-f, 4d-f, 5a-c, 5e-f are provided as a Source Data file.

Field-specific reporting

Please select the one below that is the best fit for your research. If you are not sure, read the appropriate sections before making your selection.

☒ Life sciences ☐ Behavioural & social sciences ☐ Ecological, evolutionary & environmental sciences

For a reference copy of the document with all sections, see nature.com/documents/nr-reporting-summary-flat.pdf

Life sciences study design

All studies must disclose on these points even when the disclosure is negative.

Sample size	Sample size calculations were not performed. Sample sizes for each experiment where chosen to be consistent with the field norms.
Data exclusions	No data were excluded from the experiments in this study.
Replication	Wherever possible, experimental findings were replicated both through technical replicates (stipulated as N-number in the associated legends) and independent biological replicates (stipulated as n-number in the associated legends).
Randomization	For the experiments which involved treating parent cell lines with different stimuli/inhibitors, randomization was not possible with this experimental design (as we assumed that randomizing the treatment of a parent cell line would not alter data variance). However, as stipulated in the Methods section, quantitation of immunofluorescent signals was performed on randomly captured micrographs (whereby only the Hoechst signal was visualised prior to multichannel acquisition).
Blinding	For the experiments which involved treating parent cell lines with different stimuli/inhibitors, blinding was not possible with this experimental design (as we assumed that blinding the treatment of a parent cell line would not alter data variance). Moreover, blinding was not performed for the quantitation of immunofluorescent signals as these analyses used unbiased macros which quantified all data that had been captured for each experiment.

Reporting for specific materials, systems and methods

We require information from authors about some types of materials, experimental systems and methods used in many studies. Here, indicate whether each material, system or method listed is relevant to your study. If you are not sure if a list item applies to your research, read the appropriate section before selecting a response.

Materials & experimental systems

n/a	Involved in the study	
☐	☒	Antibodies
☐	☒	Eukaryotic cell lines
☒	☐	Palaeontology
☒	☐	Animals and other organisms
☒	☐	Human research participants
☒	☐	Clinical data

Methods

n/a	Involved in the study	
☒	☐	ChIP-seq
☒	☐	Flow cytometry
☒	☐	MRI-based neuroimaging

Antibodies

Antibodies used

Primary antibodies used in this study were:
* rat anti-MLKL (clone 3H1; produced in-house by the WEHI Antibody Facility) - used extensively and specificity validated in Murphy JM et al., 2013 Immunity,
* rat anti-MLKL (clone 7G2; produced in-house by the WEHI Antibody Facility) - specificity validated in Supplementary Figure 2 and will be made publicly-available upon publication
* rat anti-MLKL (clone 10C2; produced in-house by the WEHI Antibody Facility) - specificity validated in Supplementary Figure 2 and will be made publicly-available upon publication,
* rabbit anti-phospho-MLKL phospho-S358 (Abcam #ab187091) - used extensively and specificity validated in Wang H et al., 2014 Molecular Cell,
* anti-GAPDH (Merck #MAB374) - used extensively and specificity validated as described on manufacturer's website,
* anti-VDAC1 (Merck #AB10527) - used extensively and specificity validated as described on manufacturer's website,
* anti-ZO-1 (ThermoFisher Scientific #33-9100) - used extensively and specificity validated as described on manufacturer's website,
* anti-occludin (ThermoFisher Scientific #33-1500) - used extensively and specificity validated as described on manufacturer's website,
* anti-JAM-A (Santa Cruz Biotechnology #sc-53623) - specificity validated as described on manufacturer's website,
* anti-TOMM20 (Santa Cruz Biotechnology #sc-17764) - specificity validated as described on manufacturer's website,

* anti-LAMP-2 Santa Cruz Biotechnology #sc-18822) - specificity validated as described on manufacturer's website,
* anti-Flotillin (BD Transduction Laboratories #610821) - specificity validated as described on manufacturer's website,
* anti-20S (Santa Cruz Biotechnology #sc-374405) - specificity validated as described on manufacturer's website,
* anti-HSP90a/b (Santa Cruz Biotechnology #sc-13119) - specificity validated as described on manufacturer's website,
* anti-E-cadherin (Santa Cruz Biotechnology#sc-8426) - specificity validated as described on manufacturer's website,
* anti-Desmoglein 2 (Santa Cruz Biotechnology #sc-80663) - specificity validated as described on manufacturer's website,
* rat anti-human RIPK3 (clone 1H2; produced in-house by the WEHI Antibody Facility) - used extensively and specificity validated in Petrie EJ et al., 2019 Cell Reports,
* anti-phospho-RIPK3 phospho-S227 (Abcam #ab 209384) - specificity validated as described on manufacturer's website,
* anti-RIPK1 (Cell Signaling Technology # 3493) - specificity validated as described on manufacturer's website,
* anti-Vti1a (BD Biosciences; Cat#6112200) - specificity validated as described on manufacturer's website,
* anti-tubulin (Cytoskeleton, Inc. # ATN02) - used extensively and specificity validated as described on manufacturer's website,
* anti-actin (Abcam #ab14128; used in Fig. 7a) - used extensively and specificity validated as described on manufacturer's website,
* anti-beta-Actin (Sigma #A1987; used in Supplementary Fig. 2a) - used extensively and specificity validated as described on manufacturer's website.

Secondary antibodies used in this study were:
* donkey anti-mouse IgG (LI-COR Biosciences #925-32212),
* donkey anti-rabbit IgG (LI-COR Biosciences #925-32213),
* goat anti-rat IgG (LI-COR Biosciences #925-68029),
* horseradish peroxidase (HRP)-conjugated goat anti-rat IgG (Southern Biotech #3010-05),
* HRP-conjugated goat anti-mouse IgG (Southern Biotech #1010-05),
* HRP-conjugated goat anti-rabbit IgG (Southern Biotech #4010-05).
* AlexaFluor647-conjugated donkey anti-rabbit IgG (ThermoFisher Scientific #A31573),
* AlexaFluor568-conjugated donkey anti-rabbit IgG (ThermoFisher Scientific #A10042),
* AlexaFluor568-conjugated donkey anti-mouse IgG (ThermoFisher Scientific #A10037),
* AlexaFluor488-conjugated donkey anti-rat IgG (ThermoFisher Scientific #A21208).

Validation

Further details about validation of the following commercial primary antibodies:
* anti-GAPDH (Merck #MAB374) - validated by manufacturer for use in ELISA, IP, IC, IF, IH & WB,
* anti-b-Actin (Sigma #A1987) - validated by manufacturer for use in IHC, IF & WB,
* anti-VDAC1 (Merck #AB10527) - validated by manufacturer for use in WB,
* anti-ZO-1 (ThermoFisher Scientific #33-9100) - validated by manufacturer for use in ELISA, IF & WB,
* anti-occludin (ThermoFisher Scientific #33-1500) - validated by manufacturer for use in IHC, IF & WB,
* anti-JAM-A (Santa Cruz Biotechnology #sc-53623) - validated by manufacturer for use in IF, IP & WB,
* anti-TOMM20 (Santa Cruz Biotechnology #sc-17764) - validated by manufacturer for use in ELISA, IHC, IF, IP & WB,
* anti-LAMP-2 (Santa Cruz Biotechnology #sc-18822) - validated by manufacturer for use in IHC, IF, IP & WB,
* anti-Flotillin (BD Transduction Laboratories #610821) - validated by manufacturer for use in IF & WB,
* anti-20S (Santa Cruz Biotechnology #sc-374405) - validated by manufacturer for use in ELISA, IHC, IF, IP & WB,
* anti-HSP90a/b (Santa Cruz Biotechnology #sc-13119) - validated by manufacturer for use in ELISA, IHC, IF, IP & WB,
* anti-E-cadherin (Santa Cruz Biotechnology#sc-8426) - validated by manufacturer for use in ELISA, IHC, IF, IP & WB,
* anti-Desmoglein 2 (Santa Cruz Biotechnology #sc-80663) - validated by manufacturer for use in IHC, IF, IP & WB,
* anti-phospho-RIPK3 phospho-S227 (Abcam #ab 209384) - validated by manufacturer for use in ELISA, dot blot & WB,
* anti-RIPK1 (Cell Signaling Technology # 3493) - validated by manufacturer for use in ELISA, IF, IP & WB,
* anti-Vti1a (BD Biosciences; Cat#6112200) - validated by manufacturer for use in IF & WB,
* anti-tubulin (Cytoskeleton, Inc. # ATN02) - validated by manufacturer for use in IF & WB,
* anti-actin (Abcam #ab14128; used in Fig. 7a) - validated by manufacturer for use in IF & WB,
* anti-b-Actin (Sigma #A1987) - validated by manufacturer for use in IHC, IF & WB.

Eukaryotic cell lines

Policy information about cell lines

Cell line source(s)

*Wild-type U937 cells were purchased from the American Tissue Culture Collection,
* Wild-type HT29 cells were provided by Mark Hampton (University of Otago),
* HCC2998, SW620, RPMI-8226, Molt-4 and Colo-205 cells were provided by Prof. Nick D. Huntington (Walter and Eliza Hall Institute of Medical Research) and were originally sourced from the NCI-60 panel (Shoemaker RH et al., 2006 Nat Rev Cancer),
* YAMC cells were provided by Prof. Robert Ramsay (Peter MacCallum Cancer Centre),
* The RIPK1-/-, RIPK3-/- and MLKL-/- HT29 cells were generated at the Walter and Eliza Hall Institute of Medical Research and have been previously reported (Petrie EJ et al., 2018 Nature Communications; Tanzer MC et al., 2017 Cell Death &Differentiation),
* HT29 cells expressing the short MLKL2 splice isoform were generated at the Walter and Eliza Hall Institute of Medical Research by the authors as described in the Methods section,

	HT29 cells expressing Monobody 37 (Mb37) were generated at the Walter and Eliza Hall Institute of Medical Research and have been previously reported (Petrie EJ et al., 2020 PNAS),<br> mTagRFP-Membrane-1-tagged HT29 cells were generated at the Walter and Eliza Hall Institute of Medical Research by the authors as described in the Methods section,<br>* mTagRFP-Membrane-1-tagged U937 cells were generated at the Walter and Eliza Hall Institute of Medical Research by the authors as described in the Methods section.
Authentication	Cell lines were not authenticated by genomic analysis, but their responses to stimuli, morphologies, and reactivity to species-specific antibodies were consistent with their designated origins.
Mycoplasma contamination	Mycoplasma testing was routinely performed on cell lines using either real-time PCR on cell pellets or via ELISA on conditioned medium and obtained negative results.
Commonly misidentified lines<br>(See ICLAC register)	No commonly misidentified lines were used in this study.
