# Supplementary material for: MLKL trafficking and accumulation at the plasma membrane control the kinetics and threshold for necroptosis
Source: Nat Commun. 2020 Jun 19;11:3151. doi: 10.1038/s41467-020-16887-1 (PMC7305196; doi:10.1038/s41467-020-16887-1)
Supplement: Supplementary file 4 — Description of Additional Supplementary Files [file 41467_2020_16887_MOESM4_ESM.pdf]

Description of Additional Supplementary Files

File name: Supplementary Movie 1
Description: Examples of RIPK1- and MLKL10C2-containing cytoplasmic clusters in HT29 cells undergoing TNF-induced necroptosis imaged via Airyscan super-resolution microscopy. Accompaniment to Fig. 2g.

File name: Supplementary Movie 2
Description: Examples of MLKL10C2 and MLKLpS358 hotspots in HT29 cells undergoing TNF-induced necroptosis imaged via 3-dimensional structured illumination microscopy. Accompaniment to Fig. 2e-f.

File name: Supplementary Movie 3
Description: Example of two HT29 cells and one U937 cell undergoing TNF-induced necroptosis imaged via lattice light sheet microscopy. Accompaniment to Fig. 4a-c and Supplementary Fig. 5d-e.

File name: Supplementary Movie 4
Description: Example of HT29 undergoing TNF-induced necroptosis where an Annexin V hotspot formed within the immediate vicinity of a MLKLpS358 hotspot as imaged via 3-dimensional confocal microscopy. Accompaniment to Fig. 4d.
