# Supplementary material for: MLKL trafficking and accumulation at the plasma membrane control the kinetics and threshold for necroptosis
Source: Nat Commun. 2020 Jun 19;11:3151. doi: 10.1038/s41467-020-16887-1 (PMC7305196; doi:10.1038/s41467-020-16887-1)

Source data for Figure 1a: Three technical replica blots of the same lysates (loaded in the same order) were probed then visualised via LICOR's Odyssey IR Imaging System as follows:

Blot #1:
1st Probe (MLKL^{3H1} in 700nm channel & MLKL^{pS358} in 800nm channel)
↓
membrane stripped
↓
2nd probe (GAPDH in 800nm channel)
↓
3rd probe (RIPK1^{pS166} in 800nm channel)

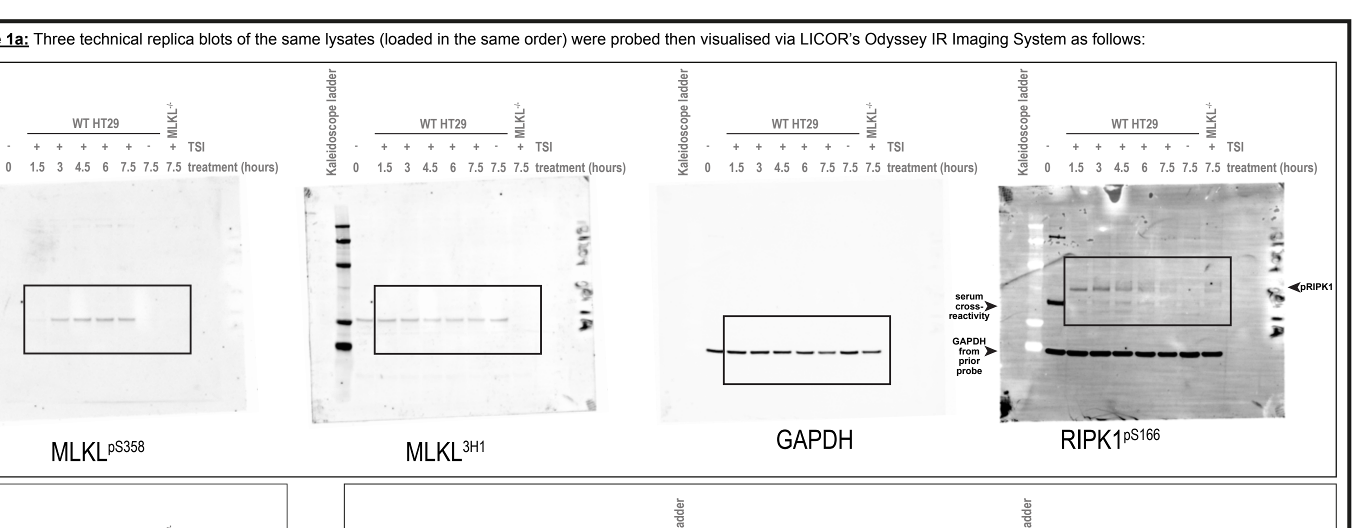

Blot #2:
1st Probe (RIPK1 in 800nm channel)

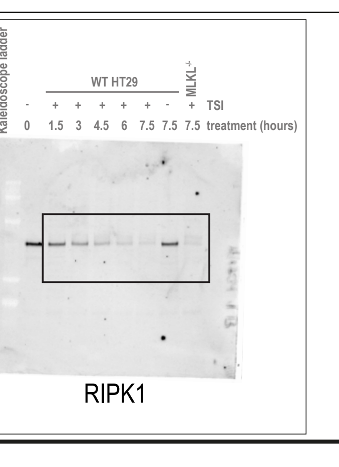

Blot #3:
1st Probe (RIPK3^{1H2} in 700nm channel & RIPK3^{pS227} in 800nm channel)

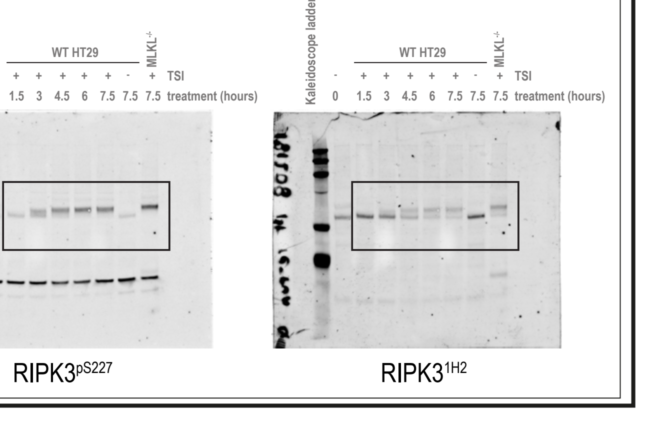

Source data underlying Figure 1b: Blots from four independent experiments were probed then visualised via LICOR's Odyssey IR Imaging System as follows:

Experiment #1
(shown in source data for Fig. 1a)

Experiment #2:
1st Probe (MLKL^{3H1} in 700nm channel & MLKL^{pS358} in 800nm channel)
↓
2nd probe (GAPDH in 800nm channel)

Experiments #3-4:
1st Probe (MLKL^{3H1} in 700nm channel & MLKL^{pS358} in 800nm channel)

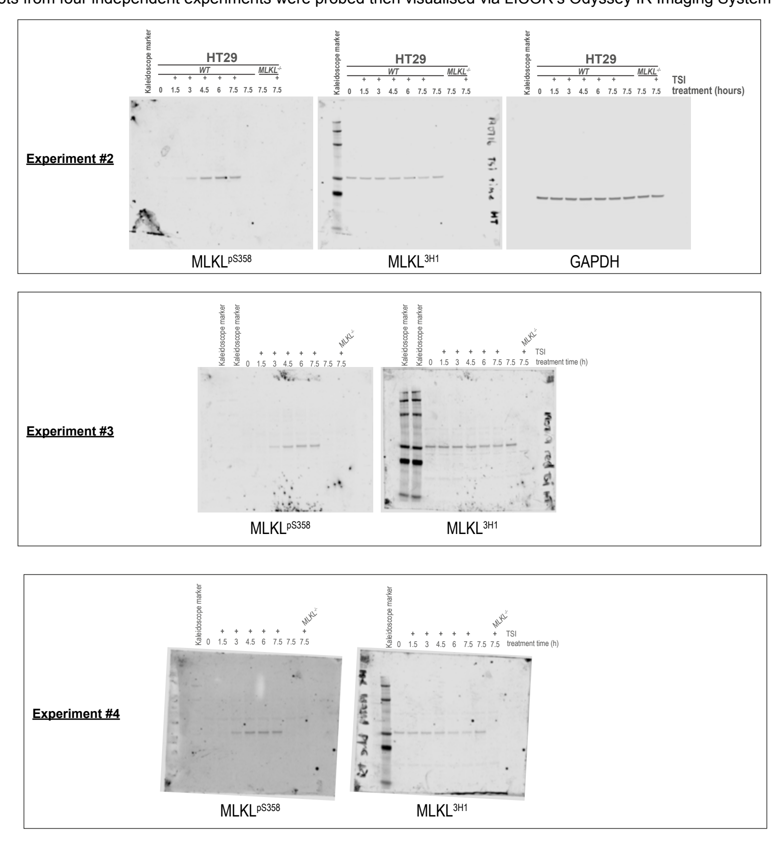

Source data for Figure 1c: Two separate blots were probed in parallel then visualised via BioRAD's Chemidoc chemiluminscence Imaging System as follows:

Blots 1&2:
1st Probe (MLKL^{3H1})
↓
membrane stripped
↓
2nd probe (MLKL^{pS358})
↓
membrane stripped
↓
3rd probe (VDAC1)
↓
4th probe (GAPDH)

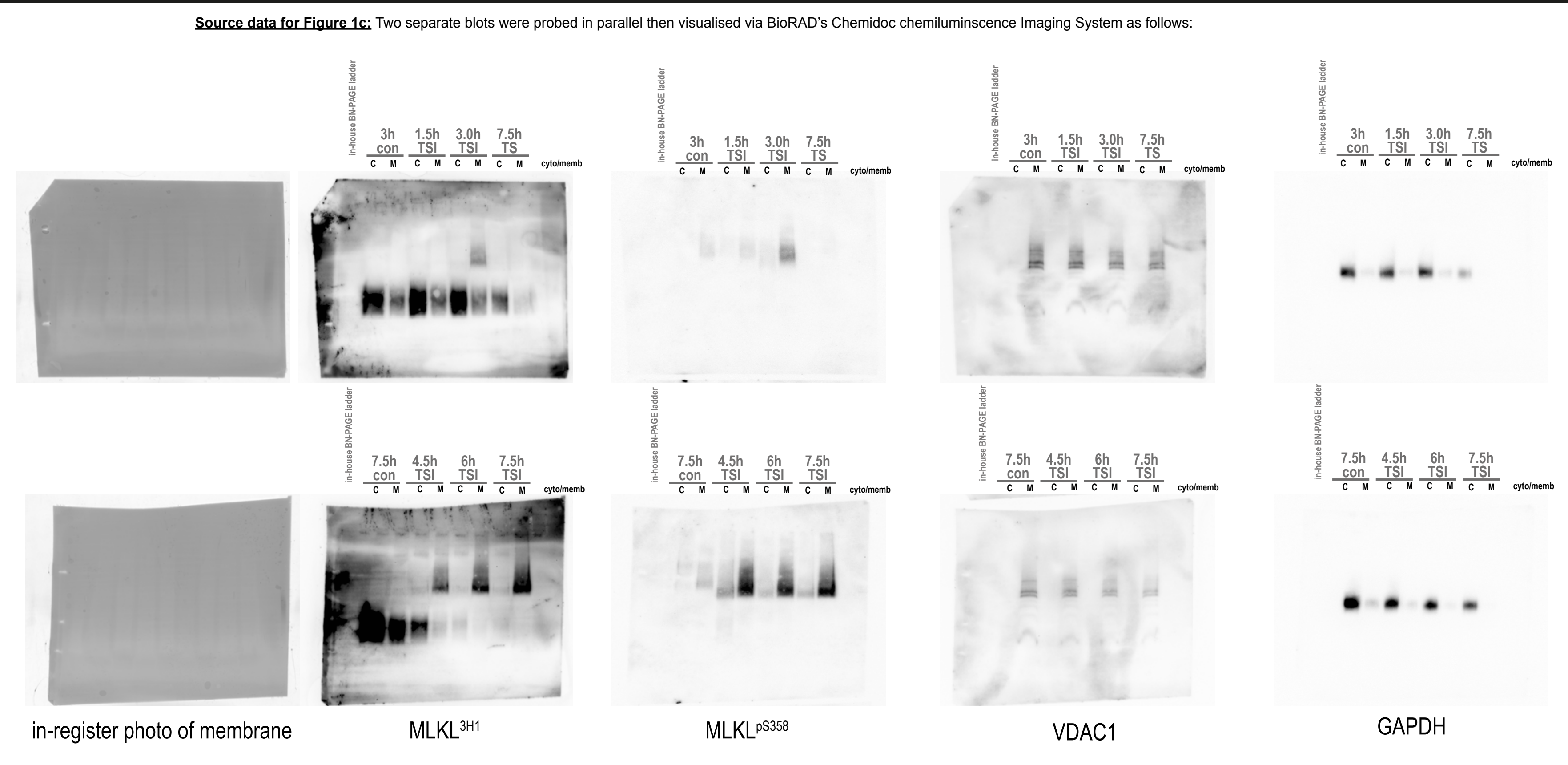

Source data for Figure 3e: Two separate blots were probed in parallel then visualised via BioRAD's Chemidoc chemiluminscence Imaging System as follows:

Blots 1&2:
1st Probe (MLKL^{3H1})
↓
membrane stripped
↓
2nd probe (MLKL^{pS358})
↓
membrane stripped
↓
3rd probe (VDAC1)
↓
4th probe (GAPDH)

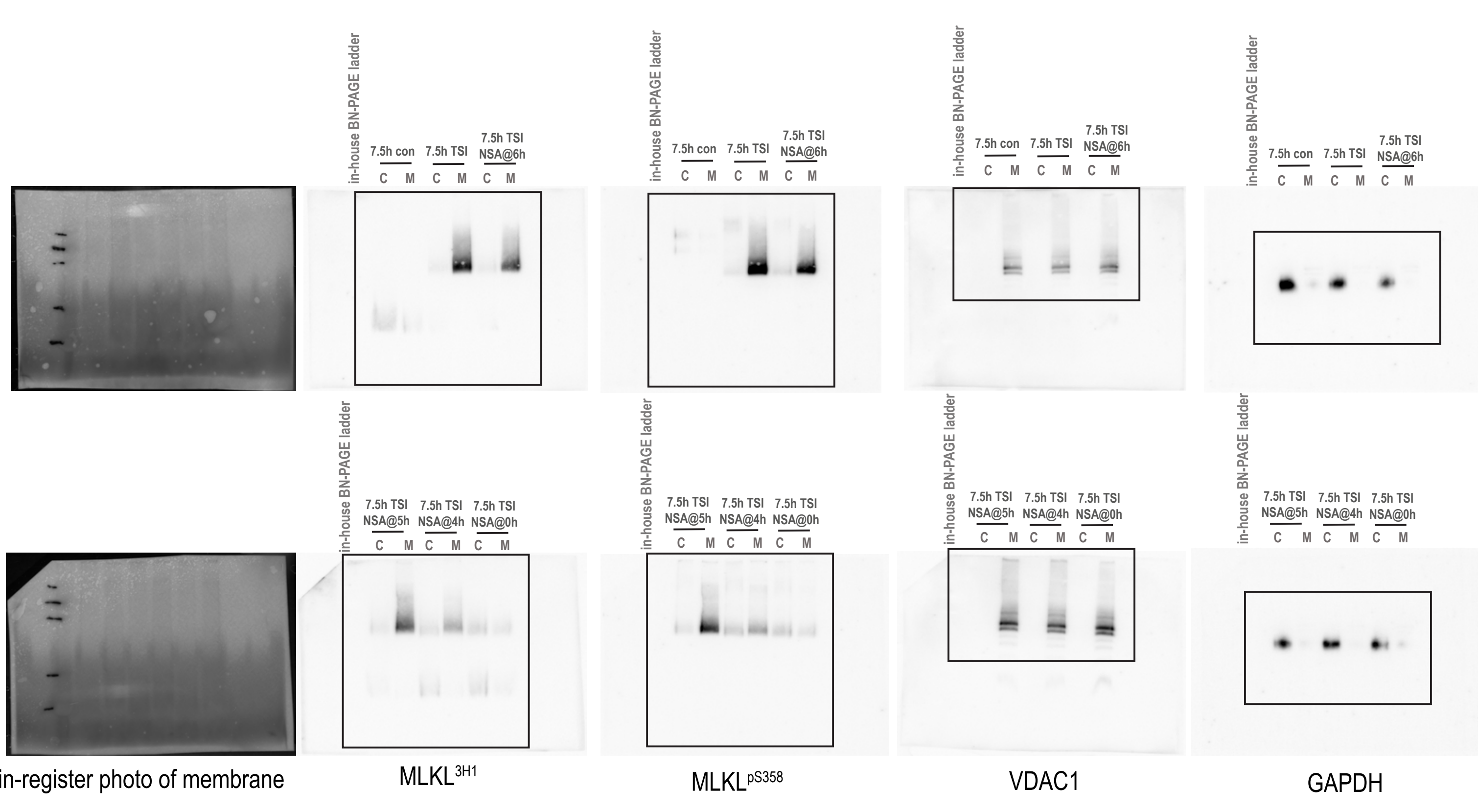

Source data for Figure 6d: Blot was probed then visualised via LICOR's Odyssey IR Imaging System as follows:

1st Probe (MLKL^{3H1} in 700nm channel & MLKL^{pS358} in 800nm channel)
↓
2nd probe (GAPDH in 800nm channel)

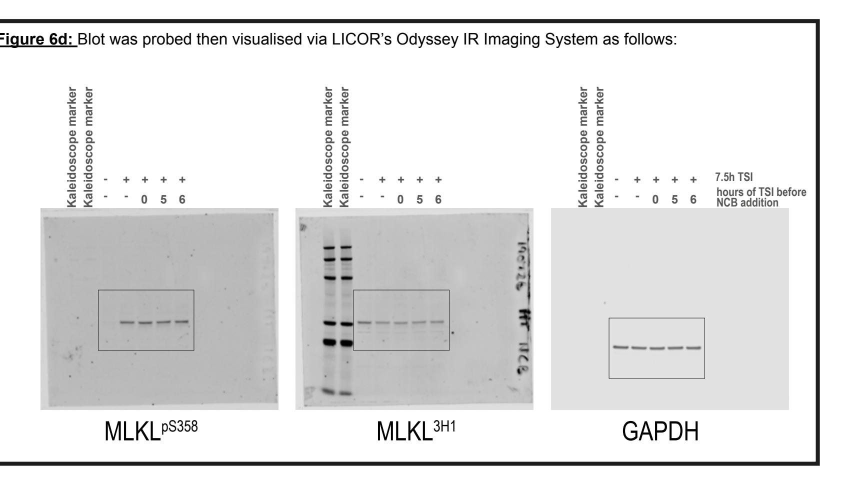

Source data for Figure 8e: Two blots from independent experiments were probed then visualised via LICOR's Odyssey IR Imaging System as follows:

1st Probe (MLKL^{3H1} in 700nm channel & MLKL^{pS358} in 800nm channel)
↓
membrane stripped
↓
2nd probe (GAPDH in 800nm channel)

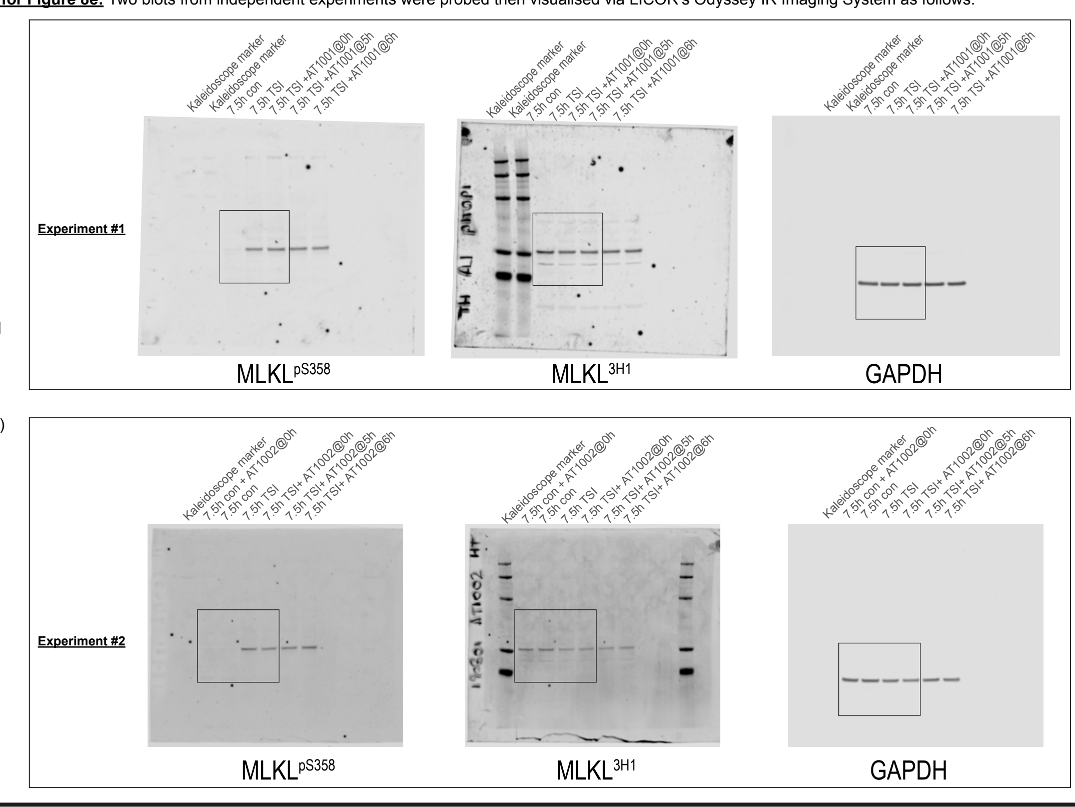

Source data for Supplementary Fig 1a,c,e: Blots were probed then visualised via LICOR's Odyssey IR Imaging System as follows:

1st Probe (MLKL^{3H1} in 700nm channel & MLKL^{pS358} in 800nm channel)
↓
membrane stripped
↓
2nd probe (GAPDH in 800nm channel)

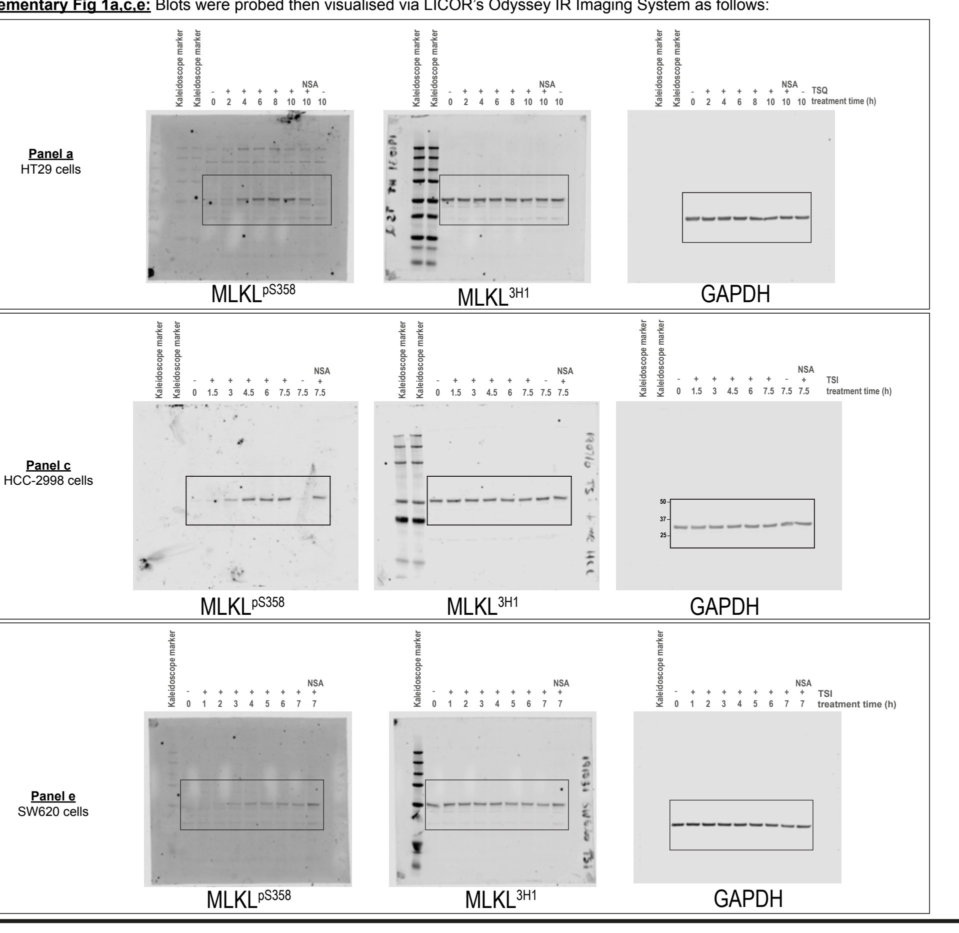

Source data for Supplementary Figure 2a: Two replica blots of the same lysates were probed in parallel then visualised via BioRAD's Chemidoc chemiluminscence Imaging System as follows:

Blots 1:
1st Probe (MLKL^{pS358})
↓
2nd probe (MLKL^{3H1})

Blots 2:
1st Probe (MLKL^{10C2})
↓
2nd probe (MLKL^{7G2})
↓
3rd probe (actin)

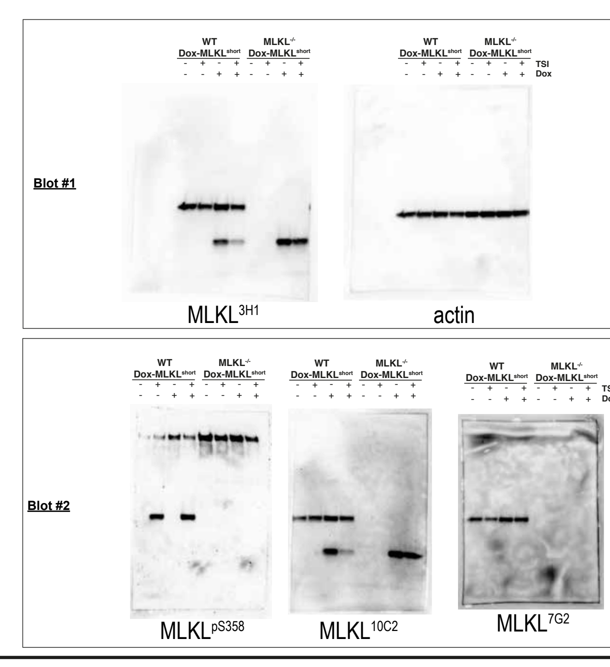

Source data for Supplementary Figure 2b: Two technical replica blots of the same purified proteins were probed then visualised via BioRAD's Chemidoc chemiluminscence Imaging System:

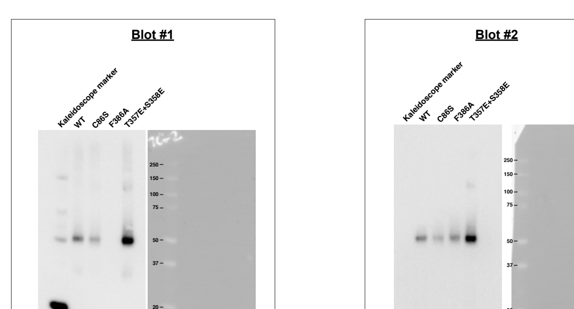

Source data for Supplementary Figure 3d: One blot was probed then visualised via BioRAD's Chemidoc chemiluminscence Imaging System as follows:

1st Probe (MLKL^{pS358})
↓
membrane stripped
↓
2nd probe (MLKL^{3H1})
↓
membrane stripped
↓
3rd probe (VDAC1)
↓
4th probe (GAPDH)

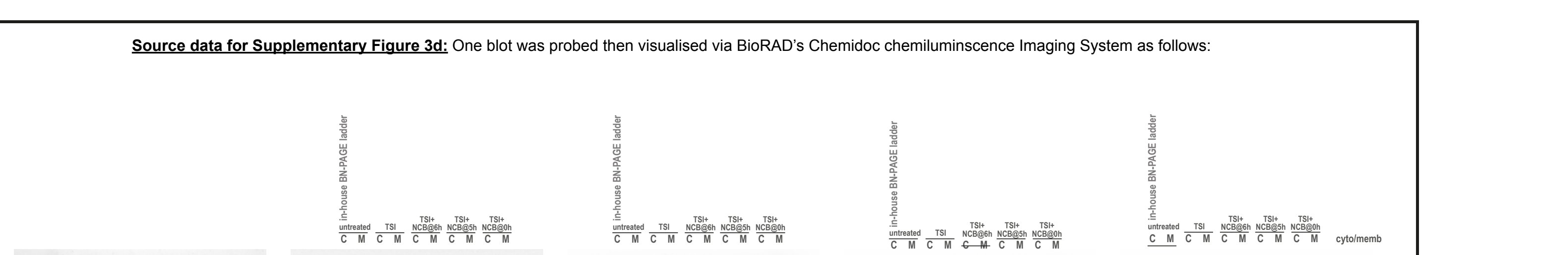

Source data for Supplementary Figure 5a: Two technical replica blots of the same cell lysates were probed then visualised via BioRAD's Chemidoc chemiluminscence Imaging System as follows:

Blot #1:
1st Probe (MLKL^{3H1})
↓
2nd probe (GAPDH)

Blot #2:
1st Probe (ZO-1)

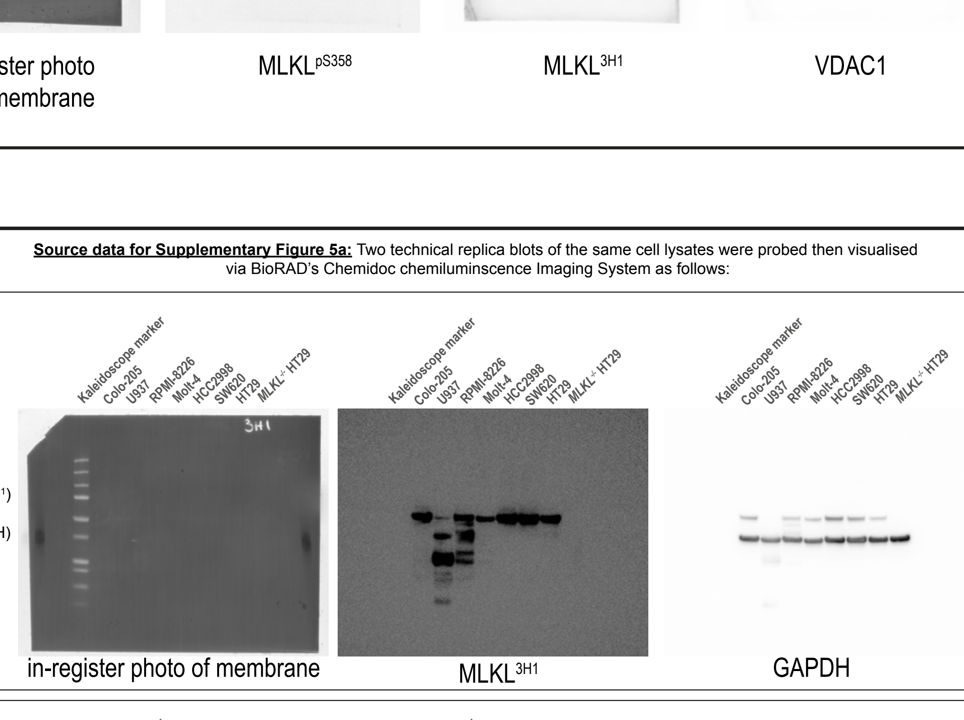

Source data for Supplementary Fig 5b: Blot was probed then visualised via LICOR's Odyssey IR Imaging System as follows:

1st Probe (MLKL^{3H1} in 700nm channel & MLKL^{pS358} in 800nm channel)
↓
membrane stripped
↓
2nd probe (GAPDH in 800nm channel)

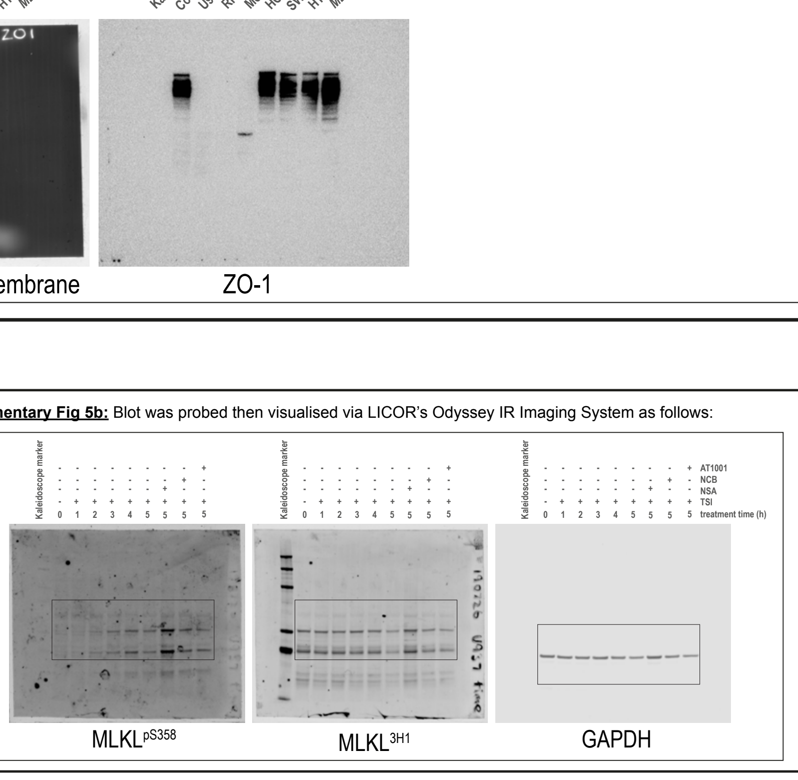

Supplement: Supplementary file 9 — Source Data [file 41467_2020_16887_MOESM9_ESM.zip › Source Data for Samson et al., 2020/200523 Source data file (blots).pdf]
