# Supplementary material for: MLKL trafficking and accumulation at the plasma membrane control the kinetics and threshold for necroptosis
Source: Nat Commun. 2020 Jun 19;11:3151. doi: 10.1038/s41467-020-16887-1 (PMC7305196; doi:10.1038/s41467-020-16887-1)
Supplement: Supplementary file 2 — Peer Review [file 41467_2020_16887_MOESM2_ESM.pdf]

Editorial Note: This manuscript has been previously reviewed at another journal that is not operating a transparent peer review scheme. This document only contains reviewer comments and rebuttal letters for versions considered at *Nature Communications.*

REVIEWERS' COMMENTS:

Reviewer #1 (Remarks to the Author):

The authors have thoroughly addressed all the points I raised either through new data or solid argument.

Reviewer #2 (Remarks to the Author):

The revised manuscript is much easier to read. While the study remains largely descriptive, it is well executed and thought-provoking. Thus, I support publication.

Reviewer #3 (Remarks to the Author):

The authors have made a good effort and addressed many of the reviewers' concerns. The manuscript has improved in quality and clarity.

The authors show that in epithelial necroptosis, MLKL assembles into hot spots at the plasma membrane and is trafficked together to tight junction proteins to the cell periphery. Accumulation of MLKL at tight junctions facilitates necroptosis propagation in neighboring cells. However, as they nicely show in this version with several new controls using different cell lines, these regulatory checkpoints are not conserved in all cell types. This is an important issue and the authors should be careful with the generalizations they made along the manuscript, based on their data.

A couple of points need to be solved still, which do not necessarily require additional experimental work:

-In the abstract the author mention that they detect MLKL in cytosolic clusters that they call necrosomes. This is however not supported sufficiently by the evidence provided and should be removed to avoid confusions.

- The temporal evolution of individual puncta into clusters and hot spots is not actually demonstrated as individual structures are not tracked over time. Moreover, MLKL translocation to internal membranes is also not shown and evidence about MLKL association with Golgi is still missing. Then, the sequence of events depicted in figure 2b is still speculative and the mechanism of vesicle formation quite unclear, which should be reflected in the text.

-Throughout the manuscript the authors repeat that the MLKL pS358 emerging from the clusters is associated with vesicles. However, they do not provide any evidence for the presence of absence of membranes in this form of MLKL neither in the one of the clusters reactive to 10C2. This reference to membranes should be removed, as it does not really affect any of the main points of the article. Perhaps it could be included as a discussion point in the discussion section if at all.

-The architecture of MLKL in intercellular junctions is more like a ring/circumference or like a circle?

-The authors associate the clusters stained with Annexin V with regions of membrane damage. However, what Annexin V stains is actually PS exposed to the outer leaflet. Unless proven otherwise, PS is a diffusing lipid that will spread on the cell surface as it reaches it. As a consequence, to my understanding, and unless the authors provide further evidence, it cannot be stated that the signal of Annexin V localizes the sites of membrane damage with spatial precision, although it is true that the binding to the cell is a proxy that there is damage somewhere in the membrane.

-As a consequence, the link between MLKL hotspots, Annexin V binding and membrane damage is only correlative at best. The sentence "These data implicate 168 MLKLpS358 hotspots as the primary source of membrane damage during epithelial cell necroptosis" or the one stating that the hotspots are the primary site of membrane damage, should be toned down (yet discussed, because it is indeed highly interesting).

-The colocalization with ZO-1 is far from obvious: most of the signals in Fig 5a do not overlap. What is the value of the pearson coefficient? The authors should quantify the % of the MLKL and the ZO-1 spots that are co-localizing and not co-localizing with each other.

-While the accumulation of MLKL at intercellular junctions seems to accelerate necroptosis in neighboring cells, the promoter of the junctional organization of ZO-1, AT-1001, reduces necroptosis and increases MLKL hotspots, while the inhibitor of junctions AT-1002 increased cell death and reduced the hotspots. This is counterintuitive given then pro-death role that the authors give to MLKL in junctional hotspots. The authors should provide an explanation and include it in the manuscript.

Reviewer #3

The authors have made a good effort and addressed many of the reviewers' concerns. The manuscript has improved in quality and clarity.

The authors show that in epithelial necroptosis, MLKL assembles into hot spots at the plasma membrane and is trafficked together to tight junction proteins to the cell periphery. Accumulation of MLKL at tight junctions facilitates necroptosis propagation in neighboring cells. However, as they nicely show in this version with several new controls using different cell lines, these regulatory checkpoints are not conserved in all cell types. This is an important issue and the authors should be careful with the generalizations they made along the manuscript, based on their data.

A couple of points need to be solved still, which do not necessarily require additional experimental work:

-In the abstract the author mention that they detect MLKL in cytosolic clusters that they call necrosomes. This is however not supported sufficiently by the evidence provided and should be removed to avoid confusions.

Response: We can replace the word "necrosomes" with "presumed necrosomes" on line 28 in the abstract. But to reiterate, our data are consistent only with these cytoplasmic MLKL clusters being necrosomal structures, because they:

- appear downstream of RIPK3-mediated MLKL phosphorylation and oligomerisation
- contain RIPK1
- do not colocalise with autophagolysosomes, mitochondria, or nuclear markers

In the absence of reagents that enable IF-detection of pRIPK1 and pRIPK3, we believe that our data strongly support the definition that the MLKL clusters are necrosome-related structures.

- *The temporal evolution of individual puncta into clusters and hot spots is not actually demonstrated as individual structures are not tracked over time. Moreover, MLKL translocation to internal membranes is also not shown and evidence about MLKL association with Golgi is still missing. Then, the sequence of events depicted in figure 2b is still speculative and the mechanism of vesicle formation quite unclear, which should be reflected in the text.*

Response: The focus of this study was endogenous MLKL and, accordingly, we have not examined tagged versions to avoid possible artefacts in localization and function being introduced. The proposed experiment would necessitate introduction of a knock-in-tagged version of MLKL to track its trafficking in live cells, which we feel is beyond the scope of this work. This would be a major undertaking, because it is clear from other studies with tagged MLKL that non-physiological artefacts emerge, such as nuclear accumulation, and thus extensive validation would be necessary.

While we agree it would be attractive to image the internal membranes with, for instance, a lipophilic dye such as DilC12, this is not technically feasible with the reagents currently available to the field. Of the dozens of antibodies that we have generated against MLKL , none can be used on paraformaldehyde-fixed cells. We can only visualise endogenous MLKL using immunofluorescence on methanol-fixed cells. Because methanol is a delipidation step, it is therefore not technically feasible to visualise internal membranes with lipophilic dyes. We expect our work will provoke further interest in this area and these technical issues to be resolved with additional, extensive experimentation.

-Throughout the manuscript the authors repeat that the MLKL pS358 emerging from the clusters is associated with vesicles. However, they do not provide any evidence for the presence of absence of membranes in this form of MLKL neither in the one of the clusters reactive to 10C2. This reference to membranes should be removed, as it does not really affect any of the main points of the article. Perhaps it could be included as a discussion point in the discussion section if at all.

Response: We disagree. Preventing MLKL's membrane association with NSA or Mb37 completely abrogates the formation of the MLKL^{pS358} puncta. These puncta also often co-stain for ZO-1 which is widely-accepted to be a membrane-associated protein. On these bases, we stand by our hypothesis that the MLKL-containing puncta are likely to be vesicles.

-The architecture of MLKL in intercellular junctions is more like a ring/circumference or like a circle?
Response: Both. Some hotspots are rings, and some circles. This is exemplified in Suppl Video 2. We have been clear about the heterogeneous nature of these structures in the text but in light of the reviewer query have reworded slightly on line 124.

-The authors associate the clusters stained with Annexin V with regions of membrane damage. However, what Annexin V stains is actually PS exposed to the outer leaflet. Unless proven otherwise, PS is a diffusing lipid that will spread on the cell surface as it reaches it. As a consequence, to my understanding, and unless the authors provide further evidence, it cannot be stated that the signal of Annexin V localizes the sites of membrane damage with spatial precision, although it is true that the binding to the cell is a proxy that there is damage somewhere in the membrane.
Response: In general, PS can diffuse laterally across membranes. However, within membrane microdomains (e.g. within nascent tight junctions), it is likely that the diffusion of externalised lipid is restricted (as mentioned in review by Zihni et al., 2016 Nat Rev Mol Cell Biol). This is one possible explanation for why, in our LLSM datasets, the Annexin V^{+} hotspots that form during necroptosis stable and do not lead to widespread and equivalent Annexin V binding across the cell membrane. Nonetheless, while the reviewer raises a good point, we can only report and interpret what we have observed in our experiments. This reviewer query is also captured in the next point, which we have clarified with some rewording.

-As a consequence, the link between MLKL hotspots, Annexin V binding and membrane damage is only correlative at best. The sentence "These data implicate 168 MLKLpS358 hotspots as the primary source of membrane damage during epithelial cell necroptosis" or the one stating that the hotspots are the primary site of membrane damage, should be toned down (yet discussed, because it is indeed highly interesting).
Response: Yes, we agree; we have reported these observations as correlative and the reviewer would prefer us to be more circumspect with our interpretation. Accordingly, we have emphasised the correlative nature of this observation both in the results and discussion to ensure that this result is not overinterpreted. We have amended the text on lines 161-165 in light of this and the preceding query.

-The colocalization with ZO-1 is far from obvious: most of the signals in Fig 5a do not overlap. What is the value of the pearson coefficient? The authors should quantify the % of the MLKL and the ZO-1 spots that are co-localizing and not co-localizing with each other.
Response: As stated in our paper, and indeed by Reviewer 1, the colocalization between ZO-1 and MLKL^{pS358} is compelling. The functional crosstalk between TJs and MLKL further speaks to this. While we believe our 2D scatterplot in Fig. 8b provides the best (most rigorous) analysis of this colocalization, we have now included Pearson's correlation coefficient of determination analysis (new Fig. 8c). This analysis further measures the degree of colocalization and indicates that 51% of MLKL^{pS358} can be explained by a linear relationship with ZO-1; which supports our initial conclusion. We have included additional text in lines 266-7 to further qualify the colocalization data.

-While the accumulation of MLKL at intercellular junctions seems to accelerate necroptosis in neighboring cells, the promoter of the junctional organization of ZO-1, AT-1001, reduces necroptosis and increases MLKL hotspots, while the inhibitor of junctions AT-1002 increased cell death and reduced the hotspots. This is counterintuitive given then pro-death role that the authors give to MLKL in junctional hotspots. The authors should provide an explanation and include it in the manuscript.
Response: We have further explained our hypothesis as to how modulating TJ trafficking, in turn, modulates the threshold for necroptosis. Simply stated, we propose that augmenting the structure of tight junctions with AT-1001 increases membrane rigidity to raise the threshold for pMLKL-directed

permeabilization. Conversely, weakening the rigidity of the membrane at tight junctions with AT-1002 will lower the threshold for lytic death. We have added text to clarify this reasoning in the revised version (lines 278-282).
